# Evaluation of the accuracy of established patient inspiratory effort estimation methods during mechanical support ventilation

**DOI:** 10.1016/j.heliyon.2023.e13610

**Published:** 2023-02-10

**Authors:** A. van Diepen, T.H.G.F. Bakkes, A.J.R. De Bie, S. Turco, R.A. Bouwman, P.H. Woerlee, M. Mischi

**Affiliations:** a Department of Electrical Engineering, Technische Universiteit Eindhoven, De Zaale, Eindhoven, 5612AZ, Noord-Brabant, the Netherlands; b Catharina Hospital, Michelangelolaan 2, Eindhoven, 5623 EJ, Noord-Brabant, the Netherlands

**Keywords:** Pressure support ventilation, Work of breathing, Pressure-time product, Inspiratory effort

## Abstract

There is a clinical need for monitoring inspiratory effort to prevent lung- and diaphragm injury in patients who receive supportive mechanical ventilation in an Intensive Care Unit. Different pressure-based techniques are available to estimate this inspiratory effort at the bedside, but the accuracy of their effort estimation is uncertain since they are all based on a simplified linear model of the respiratory system, which omits gas compressibility of air, and the viscoelasticity and nonlinearities of the respiratory system. The aim of this in-silico study was to provide an overview of the pressure-based estimation techniques and to evaluate their accuracy using a more sophisticated model of the respiratory system and ventilator.

The influence of the following parameters on the accuracy of the pressure-based estimation techniques was evaluated using the in-silico model: 1) the patient's respiratory mechanics 2) PEEP and the inspiratory pressure of the ventilator 3) gas compressibility of air 4) viscoelasticity of the respiratory system 5) the strength of the inspiratory effort.

The best-performing technique in terms of accuracy was the whole breath occlusion. The average error and maximum error were the lowest for all patient archetypes. We found that the error was related to the expansion of gas in the breathing set and lungs and respiratory compliance. However, concerns exist that other factors not included in the model, such as a changed muscle-force relation during an occlusion, might influence the true accuracy. The estimation techniques based on the esophageal pressure showed an error related to the viscoelastic element in the model which leads to a higher error than the occlusion. The error of the esophageal pressure-based techniques is therefore highly dependent on the pathology of the patient and the settings of the ventilator and might change over time while a patient recovers or becomes more ill.

## Graphical abstract

**Figure:**
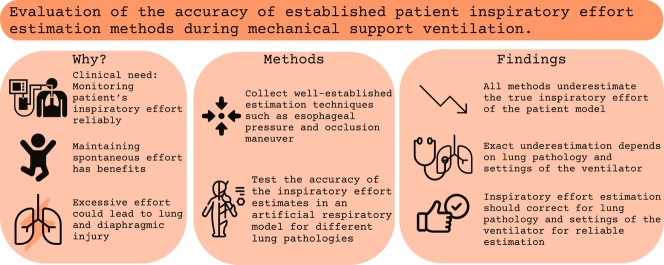

## 1 Introduction

In supportive mechanical ventilation, the patient is able to trigger the ventilator such that the ventilator can support the inspiratory effort of the patient. Both a lack and too much support from the ventilator might result in discomfort, fear, and even lung and diaphragmatic injury which can all cause stress and prolong ventilation time [1], [2], [3]. Maintaining spontaneous effort improves oxygenation, prevents atrophy of the peripheral muscles, and reduces the need for sedation [4], [5]. The inspiratory effort is also an important parameter to follow-up recovery from critical illness and is a determinant for successful weaning from the ventilator. There is a clinical need for reliable monitoring of the patient's inspiratory effort during supportive mechanical ventilation in patients in the Intensive Care, as the COVID-19 pandemic also recently demonstrated.

Various pressure-based techniques can estimate the patient's inspiratory muscle effort during supportive mechanical ventilation in clinical practice: the esophageal-derived muscle pressure, work of breathing, the pressure time product, P_0.1_, an end-expiratory occlusion test, and proportional assist ventilation (PAV) [6], [7]. Furthermore, experimental algorithms for non-invasive estimation are published such as the least square fitting method [8], constrained optimization [9], Gaussian effort model [10], b-spline effort model [11], and sparse optimization [12]; however, these methods are not clinically tested yet.

**Figure 1:**
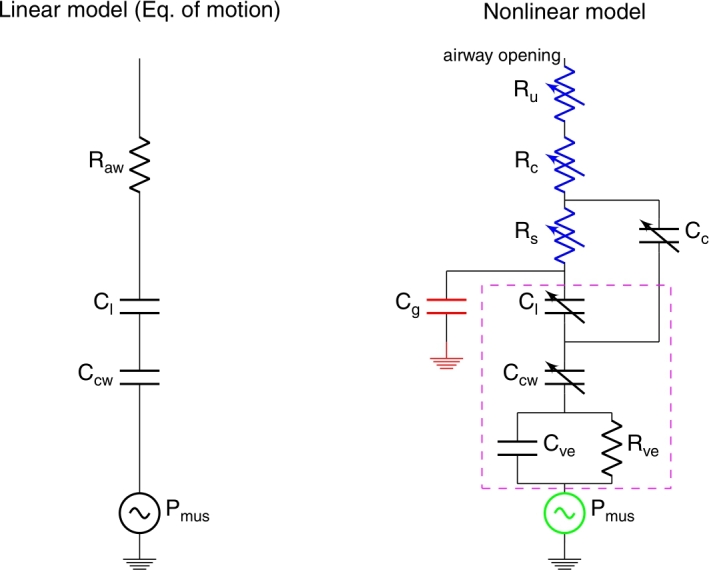
On the left, the linear model of the respiratory system from which the equation of motion is derived. On the right, the used respiratory model, a modified version of [17]. The blue resistances model the resistance of the airways. The black capacitors model the compliance of the respiratory system. The charge on the red capacitor accounts for the shift volume/gas compression (as known from full body plethysmography). The elements in the magenta box account for the viscoelastic properties of the lung-chest wall. Note that the components are modeled by nonlinear equations and might depend on each other.

All these pressure-based estimation techniques (including both clinically-used and experimental techniques) are derived from “the” equation of motion, a simplified linear lung model (Fig. 1) [13]. Although this model is widely used, it omits variables that affect the accuracy of the estimates of the patient's effort such as non-linearity of the elastance of lung and chest wall tissue, turbulence in the airway, viscoelasticity of the respiratory system (i.e. the lung, chest wall, and diaphragm) and gas compression [14], [15], [16]. These omissions and simplifications may lead to errors in the estimation of the patient effort. Furthermore, it is unclear if the strength of the inspiratory effort, positive end-expiratory pressure (PEEP) applied by the ventilator, and inspiratory pressure also affect the estimation's accuracy. Knowledge of these potential shortcomings of the estimation techniques is important for optimizing personalized lung and diaphragmatic protective ventilation.

Currently, limited data about the accuracy of these techniques are available in the literature since evaluating these techniques and the impact of the omitted variables in real-life clinical experiments is difficult. This limitation justifies a more theoretical approach. There is strong evidence in the literature that the model including viscoelasticity is correct and corresponds to clinical measurements. A part of the muscle pressure is dropped over the viscoelastic element. There is always an underestimation of the ground truth when viscoelasticity is not taken into account, the question is whether it can be neglected. Gas compression is important for the occlusion method and is also always present in the clinical case.

Using a simulation-based approach, the aim of this study was to 1) evaluate the influence of the patient's respiratory mechanics on the accuracy of the different techniques, 2) evaluate the influence of PEEP and inspiratory pressure, 3) evaluate the influence of gas compressibility of air, 4) evaluate the effect of viscoelasticity, and 5) evaluate the influence of the strength of the inspiratory effort. Quantifying the influence on these variables is important since it shows under which circumstances the inspiratory effort measurements are valid and what can be done to improve them.

**Figure 2:**
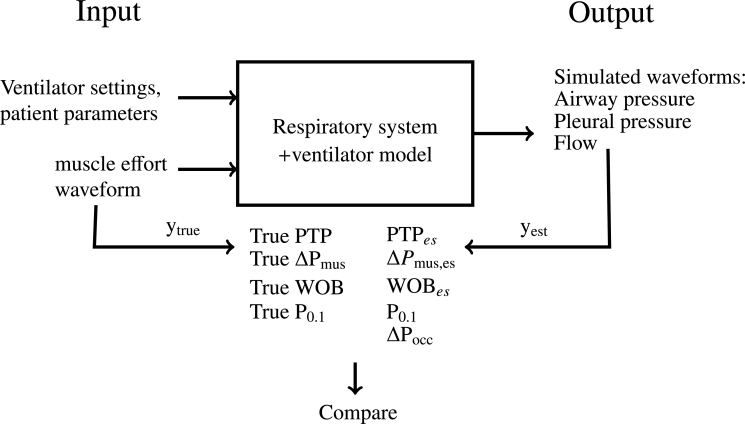
A flowchart of the approach: The ventilator settings, patient parameters, and muscle effort waveform are the input of the ventilator and respiratory model. The model generates simulated airway pressure, pleural pressure, and flow waveforms. The estimation techniques are applied to the simulated waveforms and used to calculate inspiratory effort estimates. From the input muscle effort waveform the ground truth inspiratory effort can be calculated. The estimates and ground truth are compared to investigate the accuracy of the different techniques and the impact of the different variables.

We take the following approach in this paper: We reuse a comprehensive respiratory and ventilator model, that has been published and validated before [18], [17], [19] and includes the usually omitted variables. The model takes as inputs the patient's respiratory mechanics, ventilator settings, and muscle effort waveforms and generates the airway pressure, pleural pressure, and flow during pressure support ventilation (PSV) breaths (see Fig. 2) since it is the most used spontaneous ventilation mode in the ICU. We also include PSV breaths with zero inspiratory pressure above PEEP, which is similar to continuous positive airway pressure (CPAP). The pressure-based estimation techniques are applied to the simulated waveforms, and their estimates are compared to the original input muscle waveform. By doing this, we can assess the impact of the different variables and whether they introduce an error.

## 2 Materials and methods

First, the model of the respiratory system and diaphragm model is explained in Section 2.1, and the ventilator model in Section 2.2. Next, the clinically-available pressure-based estimation techniques are discussed in more detail in Section 2.3. The protocol used to generate simulations using these models is explained in Section 2.4. Finally, the metrics used to assess the quality of the estimates of the estimation techniques are explained in Section 2.5.

### 2.1 Lung and diaphragm model

Various models of the respiratory system are available. By far the most used model is the linear one-compartment model from which the equation of motion is derived. We have chosen to use a more elaborate version of this model, which is a modified variant of the model in Liu et al. [17] and is previously described and validated in [20], [19]. The model is shown on the right in Fig. 1.

Instead of one constant resistance in the linear model, the chosen respiratory model consists of three variable resistances that model: 1) the upper airways ($R_{u}$), modeled by a so-called Rohrer resistance to account for turbulence, 2) collapsible airways ($R_{c}$) which is inversely proportional to the air volume in the collapsible airway, and 3) small airways ($R_{s}$), which depends on the volume of air in the lung.

The volume of the chest wall ($V_{\mathrm{cw}}$), lung volume ($V_{a}$) and collapsible airway segment ($V_{c}$) are modeled with a sigmoidal relation, depending on the transmural pressures over the element (see Table 1). This corresponds well with the pressure-volume relationships that are described in the literature for the lung and chest wall [15].

**Table 1:** The model equations of the respiratory system.

	Equation	Model parameters
*R*_*u*_	*A*_*u*_ + *K*_*u*_flow	*A*_*u*_, *K*_*u*_
*R*_*c*_	$K_{c}{\left(V_{\mathrm{cmax}}/V_{c}\right)}^{2}$	*K*_*c*_, *V*_cmax_
*R*_*s*_	$A_{s}e^{K_{s}(V_{A}-\mathrm{RV})/(V^{⁎}-\mathrm{RV})}+B_{s}$	*A*_*s*_, *K*_*s*_, RV, *B*_*s*_, *V*^⁎^
*V*_*cw*_	$\frac{\mathrm{TLC}-\mathrm{RV}}{0.99+\exp\frac{-(P_{\mathrm{cw}}-A_{\mathrm{cw}})}{B_{\mathrm{cw}}}}+\mathrm{RV}$†	TLC, RV, *A*_*cw*_, *B*_*cw*_
*V*_*c*_	$\frac{V_{\mathrm{cmax}}}{{\left(1+e^{-A_{c}(P_{c}-B_{c})}\right)}^{D_{c}}}$†	*V*_cmax_, *A*_*c*_, *B*_*c*_, *D*_*c*_
*V*_*A*_	$\frac{A_{l}}{\left(1+e^{-B_{l}(P_{a}-D_{l})}\right)}$†	*A*_*l*_, *B*_*l*_, *D*_*l*_
*C*_*g*_	FRC/970	–

† $P_{a}$, $P_{c}$, $P_{cw}$ are the pressures over the lung compliance, collapsible airway compliance, and chest wall compliance, respectively.

The viscoelastic properties of the respiratory system are modeled by a linear-solid model. It is formed by a constant resistance, $R_{\mathrm{ve}}$, and a constant compliance, $C_{\mathrm{ve}}$, in series with the lung-chest wall compliances (Fig. 1). This is a common method to model the viscoelastic behavior of tissue.

Airflow needs pressure generation, and according to Boyle's law, pressure generation means that a mass of air is compressed or decompressed relative to its equilibrium volume at atmospheric pressure. This leads to Equation (1).

(1) $$P_{\mathrm{atm}}V_{\mathrm{atm}}=(P_{\mathrm{atm}}+\Delta P)(V_{\mathrm{atm}}+\Delta V)\;,$$

where $P_{\mathrm{atm}}$ is the absolute atmospheric pressure (970 cmH_2_O is the absolute atmospheric pressure minus water vapor pressure), $V_{\mathrm{atm}}$ is the volume of air in the lung at atmospheric pressure, $P_{\mathrm{atm}}+\Delta P$ is the absolute pressure in the lung, and $V_{\mathrm{atm}}+\Delta V$ is the volume of the same mass of air in the lung. Assuming $P_{\mathrm{atm}}$ is much larger than Δ*P* (970 cmH_2_O vs +/- 0-50 cmH_2_O), the equation leads to the solution given by Equation (2).

(2) $$\Delta V=\frac{-\Delta PV_{\mathrm{atm}}}{P_{\mathrm{atm}}}\;.$$

Δ*V* is often referred to as ‘shift volume’ [21], [16], which is one of the driving forces during spontaneous breathing and used during full body plethysmography to measure the total lung volume. In the electrical equivalent model, this can be accounted for by adding the compliance ($C_{g}$) between the alveolar space and atmospheric pressure (Fig. 1). $C_{g}$ is then equal to $V_{\mathrm{atm}}/P_{\mathrm{atm}}$, where we assume that $V_{\mathrm{atm}}$ is equal to the functional residual capacity (FRC) and $P_{\mathrm{atm}}$ is 970 cmH_2_O. The charge on $C_{g}$ is then equal to the shift volume Δ*V*.

The model equations are summarized in Table 1. The values of the model parameters in Table 1 are given in the supplementary material. Normal, obese, and ARDS parameter sets are available and include inter-patient variability by the availability of four different parameter sets per pathology.

In the model, a pressure source, $P_{\mathrm{mus}}$, simulates the total pressure generated by the respiratory muscles and serves as a ground truth for our comparison. Various shapes of this muscle effort source are proposed in the literature to simulate spontaneous breathing patients [22]. A rounded trapezoid with a different slope for the rising edge and the falling edge is used, which is common in the literature. For the purpose of this study, we keep the rise and fall time equal while varying the depth of the muscle waveform (see Section 2.4 for the precise values).

### 2.2 Model for the ventilator

**Figure 3:**
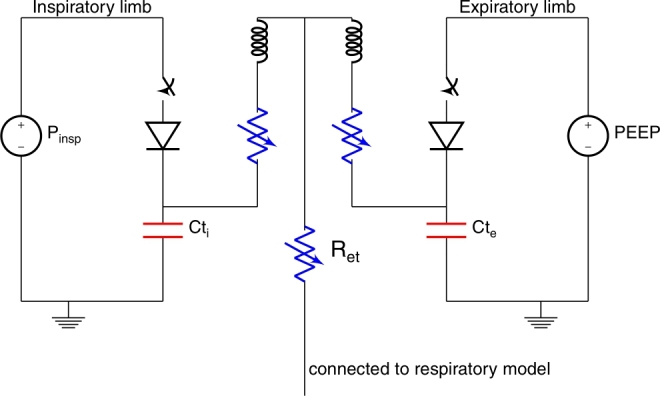
The lumped element model of the ventilator. The node labeled ‘connected to respiratory model’ is connected to the node labeled ‘airway opening’ of the respiratory model in Fig. 1. The blue resistances are Rohrer resistances.

A simplified lumped element model of a ventilator is used to simulate mechanical ventilation breaths (see Fig. 3). This simplified ventilator model was previously used to generate high-quality computer-simulated PSV and CPAP breaths [19]. The variable Rohrer resistance, $R_{et}$, accounts for the resistance of the endotracheal tube, which increases with increasing flow. The parameters of the Rohrer resistance are obtained from the manufacturer and the literature [23]. The breathing set that connects the endotracheal tube to the pressure ports on the ventilator consists of an inspiratory and expiratory circuit. Based on the details provided by the manufacturer and the literature [24], each circuit is modeled with an inductor, capacitor, and Rohrer resistance. The exact values are provided in the supplementary material.

The pressure sources in the ventilator are imitated by two voltage sources. The pressure applied to the expiratory tube is the positive end-expiratory pressure (PEEP) and the pressure applied to the end of the inspiratory tube is the inspiratory pressure ($P_{\mathrm{insp}}$). Switches and unidirectional diode-like elements model the valves in the ventilator, which block inspiration from the expiratory connection. The inspiratory valve is opened when the ventilator is triggered by the patient, and the expiratory valve is opened when the ventilator cycles. For flow triggering, a small bias flow circuit is added to the inspiratory circuit.

### 2.3 Pressure-based estimation techniques

Currently, clinically-available effort estimation techniques are all derived from the linear equation of motion. The equation of motion is derived from a linear lumped element model of the lung (Fig. 1).

#### 2.3.1 Esophageal pressure ($P_{\mathrm{es}}$)-derived muscle pressure ($P_{\mathrm{mus}}$)

**Figure 4:**
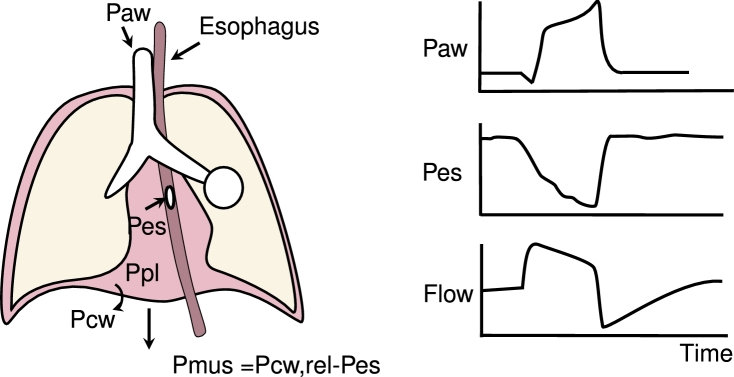
On the left, the placement of the esophageal balloon catheter in the esophagus and the derivation of the muscle pressure. On the right, the typical shapes of the airway pressure (*P*_aw_), esophageal pressure (*P*_es_), and flow during a pressure-supported breath.

This technique relies on the assumption that the pressure generated by the patient's respiratory muscles ($P_{\mathrm{mus}}$) is a surrogate for the effort of the patient. A balloon catheter is inserted through the nose or mouth and positioned in the mid-lower third of the intrathoracic esophagus behind the heart. The esophageal wall touches the outside pleura in this position which makes the measured esophageal pressure proportional to the pleural pressure (Fig. 4) [25], [26], [27]. Inspiratory effort causes a negative swing in pleural pressure, and changes in the esophageal pressure ($\Delta P_{\mathrm{es}}$) can therefore be used to track changes in the inspiratory effort (Fig. 4). The effect of the muscle effort on the esophageal pressure is attenuated by the chest wall, because of this, the swings in the esophageal pressure are an attenuated surrogate of the swing in $P_{\mathrm{mus}}$. An estimate $P_{\mathrm{mus}}$ can be calculated by subtracting $\Delta P_{\mathrm{es}}$ from the static recoil pressure of the chest wall ($\Delta P_{\mathrm{cw},\mathrm{rel}}$) which can be calculated with data from the ventilator and the chest wall compliance [25], [28]:

(3) $$\Delta P_{\mathrm{mus},\mathrm{pes}}=\Delta P_{\mathrm{cw},\mathrm{rel}}-\Delta P_{\mathrm{es}}\;.$$

The advantage of this technique is that it is relatively easy to interpret the pressure; in the simplified linear model, this technique is the exact pressure generated by the muscles. The esophageal pressure is a continuous measurement, and it is, therefore, possible to calculate $\Delta P_{\mathrm{mus},\mathrm{pes}}$ for every breath.

The disadvantages are a risk of complications when using an invasive technique, the need for a specialist who can place the catheter in the esophagus and interpret the results, extra costs, and the concern that the measured changes in esophageal pressure only reflect the local behavior of the lung due to lung inhomogeneities instead of the global pleural pressure as a surrogate for inspiratory effort [29]. Furthermore, the chest wall compliance may change unpredictably in critically ill patients with the application of PEEP which makes the estimation of $\Delta P_{\mathrm{cw},\mathrm{rel}}$ incorrect. Likewise, the viscoelasticity of the respiratory system is not taken into account which might lead to an underestimation of the muscle pressure. Lastly, $\Delta P_{\mathrm{mus},\mathrm{pes}}$ only evaluates the swing of the inspiratory effort but does not take into account the duration of the effort.

#### 2.3.2 Pressure time product

The pressure time product (PTP_es_) is the integral of $P_{\mathrm{mus},\mathrm{pes}}$ over the inspiratory time (Equation (4)).

(4) $${\mathrm{PTP}}_{\mathrm{es}}=\int_{{\mathrm{ti}}_{\mathrm{start}}}^{{\mathrm{ti}}_{\mathrm{end}}}P_{\mathrm{mus},\mathrm{pes}}\;dt\;,$$

where ${\mathrm{ti}}_{\mathrm{start}}$ is the start of inspiration and ${\mathrm{ti}}_{\mathrm{end}}$ the end of inspiration. Ideally, the true muscle pressure ($P_{\mathrm{mus}}$) would be used, but it is instead replaced with the esophageal-derived muscle pressure $P_{\mathrm{mus},\mathrm{pes}}$.

The advantage of the pressure time product is that it also takes into account the duration of inspiration, it, therefore, has a good correlation with oxygen consumption of the respiratory muscles [30]. In addition, PTP can also take into account the respiratory rate of a patient by calculating PTP per unit of time. Since PTP does not depend on the flow, it can also be used to estimate effort during occlusions and other circumstances under which there is zero flow (e.g. during the time before triggering of the ventilator). The technique is relatively easy to understand, and is easy to calculate for modern ventilators, although it might be more difficult to manually calculate at the bedside than $\Delta P_{\mathrm{mus},\mathrm{pes}}$.

All the disadvantages related to the use of the esophageal balloon catheter also apply to the PTP technique. PTP makes use of the integral of $P_{\mathrm{mus},\mathrm{pes}}$, which means that any estimation errors in $P_{\mathrm{mus},\mathrm{pes}}$ are exacerbated.

#### 2.3.3 Work of breathing

**Figure 5:**
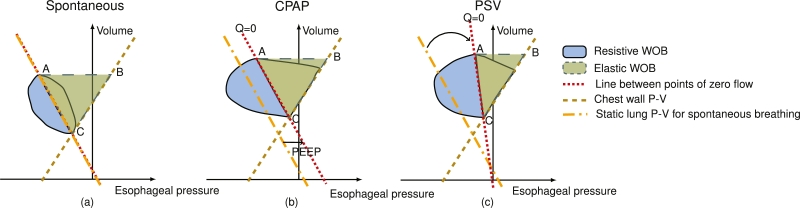
(a) shows the Campbell diagram for spontaneous breathing. The line of zero flow (‘Q=0’) coincides with the static lung pressure-volume (P-V) line here. (b) shows the diagram for CPAP. The Q=0 line is shifted by PEEP to the right. (c) shows the diagram for a pressure-supported breath. The Q=0 line is shifted due to PEEP and tilted due to the inspiratory pressure. The triangle spanned by ‘ABC’ is in all cases the elastic work of breathing. Note that for simplicity, the effect of the viscoelasticity on the esophageal pressure and active expiration are not drawn.

Work of breathing (WOB) is the energy used by the muscles for respiration. WOB can be evaluated using the Campbell diagram. In the Campbell diagram, the esophageal pressure-volume loop and the chest wall pressure-volume line are plotted. The Campbell diagram was originally developed for spontaneous breathing, and originally also used the lung pressure-volume line (Fig. 5(a)). It is extended to continuous positive airway pressure (CPAP) and pressure support ventilation (PSV) breaths by replacing the lung pressure-volume curve with a line between the points of zero flow of the esophageal pressure-volume loop [31], the ‘Q=0’ line (Fig. 5(b-c)). This line splits the inspiratory WOB (when no active expiration is present) into a resistive part and an elastic part. The elastic WOB is equal to the triangle that is enclosed by the line of zero flow, the highest volume of the lung, and the chest wall P-V line (area ABC in Fig. 5). The resistive WOB is the area left of the line of zero flow, enclosed by the line of zero flow and the esophageal pressure-volume loop (the blue area in Fig. 5). The total inspiratory WOB can then be calculated by adding the resistive WOB and elastic WOB.

The advantage of WOB is that it gives the total energy expenditure by the respiratory muscles, and is therefore useful for determining correct ventilator settings.

A downside of WOB is that it does not take into account the work that is done when no volume is displaced. It, therefore, does not correlate as well with oxygen consumption of the respiratory muscles as PTP [30]. Again, esophageal pressure must be used, and therefore all the disadvantages relating to the esophageal balloon catheter also apply here [32]. Also, an estimate of the chest wall compliance must be available and a wrong estimate could possibly lead to large errors. WOB is also difficult to calculate in real-time at the bedside by clinicians themselves, although it can easily be calculated automatically by a modern ventilator.

#### 2.3.4 End-expiratory occlusion pressure

The end-expiratory occlusion maneuver is a hold at the end of the expiration of the previous breath, just before a new inspiration effort starts (therefore called end-expiratory). The assumption is that during an occlusion, the flow in the respiratory system is close to zero. Therefore, there is no longer a pressure drop over the airway resistances and resistances of the breathing set, which would mean that the change in airway opening pressure is equal to the pressure change generated by the contraction of the respiratory muscles [33].

From the occlusion maneuver, the maximum swing in airway pressure during the entire respiratory cycle ($\Delta P_{\mathrm{occ}}$) can be extracted.

Advantages of the occlusion maneuver are that no extra invasive sensors are required, and the maneuver can be done on all ventilators by simply occluding the breathing set.

A disadvantage is that the occlusion maneuver is not a continuous measurement and therefore does not give a breath-by-breath estimate of the patient's effort. The occlusion pressure at the airway opening is influenced by the expansion of air in the lung and tubing during the occlusion maneuver, which is often neglected. A relationship can be derived based on Boyle-Mariote's law:

(5) $$\Delta P_{\mathrm{occ}}=\frac{C_{\mathrm{rs}}}{C_{\mathrm{rs}}+C_{g}+C_{\mathrm{tube}}}\Delta P_{\mathrm{mus}}$$

where $C_{\mathrm{rs}}$ represents the combined compliance of the chest wall, lung, and collapsible airways. $C_{\mathrm{tube}}$ represents the parasitic compliance of the tubing and $C_{g}$ the gas compression compliance. The occlusion pressure at the airway opening is therefore not exactly equal to the pressure drop caused by the muscles. However, if the respiratory compliance, parasitic compliance of the tubing, and gas compression compliance (based on the volume of the lung) are known, the occlusion pressure can be compensated. The force-velocity and force-length relation of the respiratory muscles are different during an occlusion [34] and concerns exist that this leads to errors. Nevertheless, a linear relation between electromyography (EMG) and occlusion pressure was found in anesthetized animals [35] and a linear relation between $\Delta P_{\mathrm{mus},\mathrm{pes}}$ and $\Delta P_{\mathrm{occ}}$ was found in men [36], [37]. End-expiratory volumes above normal (autoPEEP) could also cause a pressure loss during occlusions [33].

#### 2.3.5 P_0.1_

$P_{0.1}$ was originally introduced as the drop in airway pressure during an end-expiratory occlusion after 100 ms from the onset of the inspiratory effort. $P_{0.1}$ was introduced because conscious subjects may modify their breathing pattern unpredictably as a result of an occlusion, but usually not during the first 200 ms [33]. It was shown that the occlusion pressure and drop in pressure before triggering are similar [38]. Therefore, nowadays, some ventilators calculate $P_{0.1}$ during a regular PSV breath, by extrapolating the drop in airway pressure before triggering, and no occlusion is necessary [39]. Because of this extrapolation, $P_{0.1}$ can be calculated for every breath and is often displayed on the ventilator screen.

Disadvantages of $P_{0.1}$ are that in lungs with inhomogeneities, pendelluft could possibly influence the first part of the airway pressure which might influence $P_{0.1}$ [33]. Also, autoPEEP might cause a pressure loss, resulting in an error in $P_{0.1}$. Similar to the whole breath occlusion pressure, $P_{0.1}$ is also affected by the expansion of gas in the lung; thus, Equation (5) also holds for $P_{0.1}$.

### 2.4 Evaluation strategy

The models are simulated in LT Spice XVII [40], which is a freely available circuit simulator. Initialization is done in MATLAB R2019b [41].

The goal of the simulations was to: 1) evaluate the influence of PEEP and inspiratory pressure, 2) evaluate the influence of the inspiratory effort strength, and 3) evaluate the effect of viscoelasticity and gas compressibility. Besides, all these simulations are done for four normal archetype patients, four obese archetype patients, and four ARDS archetype patients, to assess how much the patient's pathology influences the outcomes.

To this end, multiple simulations were done, using the different patient parameter sets (obese, ARDS, and normal), different ventilator settings, and different muscle effort strengths. The used values for the muscle effort and ventilator settings are given in Table 2, the patient parameters can be found in the supplementary material.

**Table 2:** Settings for the muscle waveforms and ventilator.

Setting	Value
Muscle swing (cmH_2_O)	5, 10, 15, 20, 25 1
Rise time muscle (s)	0.5
Fall time muscle (s)	0.3
Breathing rate (breaths/min)	12
PEEP (cmH_2_O)	0, 5, 10, 15, 20
Pressure support (cmH_2_O)	PEEP+0 (CPAP),+5, +10, +15
Flow trigger (L/s)	0.05
Flow cycling (L/s)	0.35

1 Occlusions are simulated until 20 cmH_2_O due to stability issues.

In total, 1200 simulations were done with these settings. Each simulation consists of 5 breaths. The third breath is used to compare the accuracy of the pressure-based estimation techniques with the ground truth. The third breath is chosen to take into account the effect of the adjacent breaths and to get rid of start-up and end-of-breath effects.

Occlusions are simulated by closing the inspiratory and expiratory tubes close to the ventilator. The occlusion is made before the inspiratory effort starts and lasts during the whole respiratory cycle of the patient. Two PSV breaths proceed and succeed the occlusion.

### 2.5 Quality metrics

To quantify the error and compare the different measurement techniques, we report the root mean square percentage error (RMSPE), which we define in Equation (6).

(6) $$\mathrm{RMSPE}=\sqrt{\frac{1}{N}\sum_{i=1}^{N}{\left(\frac{y_{\mathrm{est},i}-y_{\mathrm{true},i}}{y_{\mathrm{true},i}}\right)}^{2}}\cdot 100\text{\%}\;,$$

where $y_{\mathrm{est}}$ is the estimated muscle effort by the measuring technique, $y_{\mathrm{true}}$ is the ground truth muscle effort input of the model and *N* the number of analyzed breaths. $y_{est}$ and $y_{true}$ depend one and are shown in Table 3.

**Table 3:** y${}_{est}$ and y${}_{true}$ used to calculate the RMSPE and PE per technique.

	y_est_	y_true_
Esophageal-derived muscle pressure	Δ*P*_mus,pes_ = max tidal volume/*C*_*cw*_ − Δ*P*_es_	Δ*P*_mus_
Pressure time product	${\mathrm{PTP}}_{\mathrm{mus},\mathrm{es}}=\int_{{\mathrm{ti}}_{\mathrm{start}}}^{{\mathrm{ti}}_{\mathrm{end}}}\mathrm{tidal volume}/C_{cw}-P_{\mathrm{es}}\;dt$	${\mathrm{PTP}}_{\mathrm{mus}}=\int_{{\mathrm{ti}}_{\mathrm{start}}}^{{\mathrm{ti}}_{\mathrm{end}}}P_{\mathrm{mus}}\;dt$
Work of breathing	As described in Section 2.3	${\mathrm{WOB}}_{\mathrm{true}}=\int_{{\mathrm{ti}}_{\mathrm{start}}}^{{\mathrm{ti}}_{\mathrm{end}}}P_{\mathrm{mus}}\cdot \mathrm{flow}\;dt$
Occlusion pressure	Δ*P*_occ_	Δ*P*_mus_
P0.1	Δ*P*_0.1,airway_	Δ*P*_0.1,mus_

In the final comparison we make use of the percentage error which we define in Equation (7)

(7) $${\mathrm{PE}}_{i}=\frac{\left|y_{\mathrm{est},i}-y_{\mathrm{true},i}\right|}{\left|y_{\mathrm{true},i}\right|}\cdot 100\text{\%}\;.$$

## 3 Results

### 3.1 Effect of PEEP and inspiratory pressure

**Figure 6:**
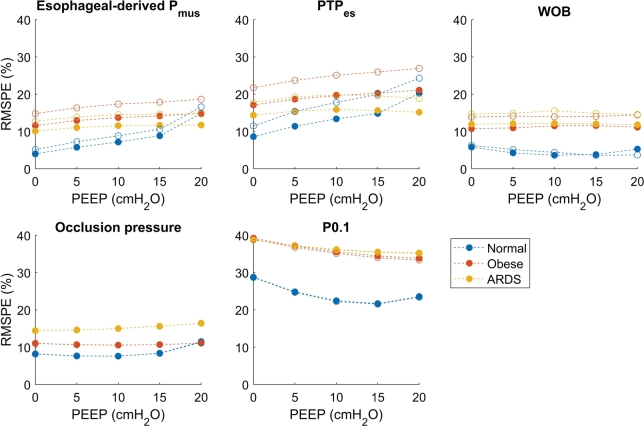
The dependency of the RMSPE on PEEP for the different techniques. Inspiratory pressure is fixed to PEEP+10 cmH_2_O (no inspiratory pressure is applied during the occlusions), *P*_mus_ is 9.9 cmH_2_O for the empty circles, and 14.85 cmH_2_O for the filled circles, and the model including viscoelasticity and gas compressibility is used.

Fig. 6 shows the dependency of the RMSPE per technique on PEEP for two inspiratory effort strengths. The RMSPE becomes higher for most techniques with increasing PEEP, except for $P_{0.1}$ and WOB, where they remain equal or become lower with increasing PEEP. The RMSPE is higher for lower patient effort, except for the occlusion pressure and $P_{0.1}$, where they mostly overlap. In all cases, the obese and ARDS archetypes have a much higher RMSPE than the normal archetype simulations. Also, the RMSPE is in all cases caused by an underestimation of the true value, however, this is not visible in the figure due to the definition of the RMSPE.

**Figure 7:**
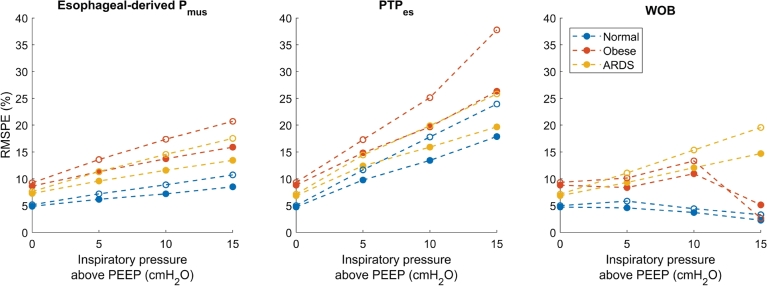
The dependency of the RMSPE on the inspiratory pressure (above PEEP) for the different techniques. PEEP is fixed to 10 cmH_2_O, *P*_mus_ is 9.9 cmH_2_O for the empty circles and 14.85 cmH_2_O for the filled circles, and the model including viscoelasticity and gas compressibility is used. 0 cmH_2_O inspiratory pressure above PEEP is CPAP.

Fig. 7 shows the dependency of the RMSPE of the different techniques on the inspiratory pressure above PEEP. During an occlusion, no inspiratory pressure is given, and therefore the occlusion pressure and $P_{0.1}$ are not reported in the figure. The RMSPE becomes higher when the inspiratory pressure is increased for the esophageal-derived $P_{\mathrm{mus}}$ and PTP. The RMSPE is highest for the obese archetype, followed by the ARDS archetype. For both techniques, the error is higher when the muscle pressure is lower. For WOB, the error first increases with increasing inspiratory pressure but then decreases for obese and normal archetype simulations. This can be explained as follows: the ‘Q=0’ line tilts right with the application of inspiratory pressure, further towards the chest wall pressure-volume line (see Fig. 5). At some point, the ‘Q=0’ line is equal to the chest wall pressure-volume line or might even lie beyond the chest wall pressure-volume line and the elastic WOB is calculated to be zero, only the error of the resistive WOB now contributes to the total error. The RMSPE is in all cases higher when the muscle pressure is lower. Again, the error is in all cases caused by an underestimation of the ground truth.

### 3.2 Effect of inspiratory effort strength

**Figure 8:**
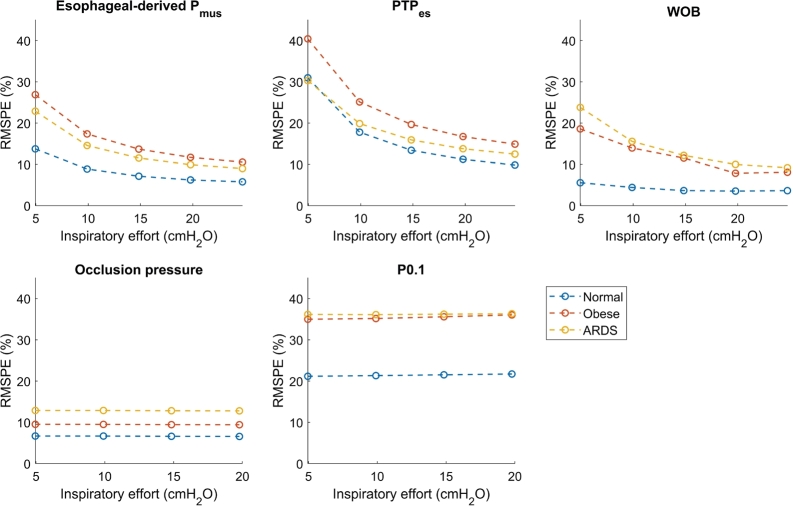
The dependency of the RMSPE on the inspiratory effort for the different techniques. PEEP is fixed to 10 cmH_2_O, inspiratory pressure above PEEP is fixed to 10 cmH_2_O (except for the occlusion), and the model including viscoelasticity and gas compressibility is used.

Fig. 8 shows the RMSPE against inspiratory effort. The techniques using the esophageal pressure (Esophageal-derived $P_{\mathrm{mus}}$, PTP, and WOB) show a decrease in RMSPE when the inspiratory effort is increased. The RMSPE of the occlusion-based techniques is independent of the strength of the inspiratory effort. Again, the error is in all cases caused by an underestimation of the ground truth.

### 3.3 Effect of viscoelasticity and gas compressibility

**Figure 9:**
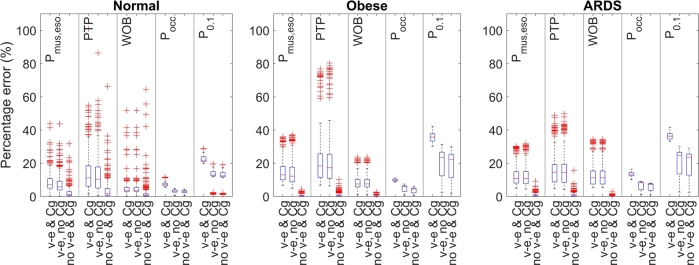
Boxplot of the percentage error per patient archetype, per technique, including and excluding viscoelasticity and gas compressibility and parasitics of the tubing. ‘v-e & Cg’ means that viscoelasticity and gas compressibility are used in the model, ‘v-e & no Cg’ means that viscoelasticity is included in the model, but gas compressibility is set to zero, and ‘no v-e & Cg’ means that both viscoelasticity and gas compressibility are not included in the model. Used PEEP: 0 (CPAP), 5, 10, 15, 20 cmH_2_O, used inspiratory pressure above PEEP: 0, 5, 10, 15 cmH_2_O, and use muscle pressure: 4.95, 9.9, 14.85, 19.78 cmH_2_O.

Fig. 9 shows a boxplot of the percentage error for normal, obese and ARDS patient archetypes per technique including and excluding viscoelasticity and gas compressibility. The estimates of the occlusion pressure, P_0.1_ and WOB all underestimate the ground truth. The esophageal-derived muscle pressure and PTP underestimate the ground truth when the visco-elasticity is included in the model, however, slightly overestimate the true value when the viscoelasticity is not included.

The figure shows that the error of the esophageal-derived muscle pressure, pressure time product, and WOB (the techniques that are based on the esophageal pressure) mainly depends on the viscoelasticity of the respiratory system since the estimation error becomes much lower when the viscoelasticity is removed from the model. Moreover, it can be seen that the error for these techniques is higher for archetypes with high viscoelasticity (the obese and ARDS archetype).

The error of the occlusion pressure and $P_{0.1}$ decreases when the gas compressibility is removed from the model. Removal of the viscoelasticity has almost no effect on the error of $P_{0.1}$ and the occlusion pressure. The error in $P_{0.1}$ is overall higher than that of the whole breath occlusion pressure. Especially the occlusion pressure has a much smaller maximum error than the other techniques, and the spread of the error is much smaller compared to the other techniques.

## 4 Discussion

In this comparative in-silico study, we evaluated the accuracy of five clinically available pressure-based techniques that estimate the patient muscle inspiratory effort during supportive mechanical ventilation with a more sophisticated non-linear model of the respiratory system. The main finding was that all techniques underestimate the true muscle effort. The secondary findings were that not taking into account of the gas compressibility and viscoelasticity can lead to substantial errors in the estimation of inspiratory effort for critically-ill patient types. Furthermore, the degree of underestimation also varies depending on the strength of the inspiratory effort, and ventilator settings, i.e. a higher error for lower inspiratory effort strengths with high inspiratory pressure above PEEP. These estimation techniques can still be used for assessing respiratory effort, especially when they are only used to observe a change in respiratory effort while the ventilator settings and respiratory mechanics remain stable. However, a possible ‘safe range’ for the maximum allowable inspiratory effort for lung- and diaphragmatic protective ventilation, should take the patient's pathology and ventilator settings into account to allow for a personalized acceptable inspiratory effort threshold. More clinical research is required to confirm this.

For the esophageal-derived muscle pressure, the error becomes higher when the level of ventilator assistance increases, when the patient's effort decreases, and when viscoelasticity increases. The error mostly disappears when the viscoelasticity is removed from the model. This can be explained by the fact that, during the measurement of the esophageal pressure, there is airflow in the respiratory system. The higher the airflow (for example by increasing the inspiratory pressure), the higher the pressure drop over the viscoelastic element will be. Since no compensation for the viscoelasticity is done when the esophageal-derived muscle pressure is calculated, the error will be higher when there is more flow in the respiratory system. For archetypes with higher viscoelasticity, such as obesity and ARDS, the error will be higher due to a higher pressure drop caused by the higher viscoelasticity during the same airflow. Although in the model this is a result of our modeling choice, several clinical studies have shown the existence of viscoelasticity and its influence on the pressure and flow waveforms [42], [43], [44]. Furthermore, it should be noted, that we used the exact chest wall compliance to calculate the esophageal-derived muscle pressure, however, if only an estimate of the chest wall compliance is available, the error is even higher. Besides this, when viscoelasticity is high, it might also lead to an underestimation of the chest wall compliance of the patient depending on how it is measured.

It is challenging to relate the observed error to previously done clinical studies since only indirect measurement of the muscle pressure is possible through either these pressure-based estimates or through the measurement of the electrical activity of the diaphragm (EAdi). However, in a clinical study [45] indeed a curvilinear relation was found between EAdi and esophageal muscle pressure when the pressure support level was increased. This implies that the coupling between EAdi and esophageal muscle pressure changes, or the esophageal muscle pressure is distorted by increasing inspiratory pressure. In [46], for high PEEP, esophageal derived-muscle pressure decreased while EAdi stayed the same as during low PEEP. This again implies that the coupling between the signals changes because of PEEP. In [37], it was found that the esophageal pressure during a PSV breath was 0.75 times the occlusion pressure, while EAdi remained the same during both breaths. The paper gave no reason for this, but it might be (partly) explained by the underestimation of the esophageal-derived muscle pressure. The change in coupling in the papers could possibly be explained by a higher pressure drop due to viscoelastic losses; however, more clinical research should be done to confirm this.

The pressure time product is the integral of the esophageal-derived muscle pressure. Therefore, any error that is made in the estimation of the esophageal-derived muscle pressure is exacerbated in the estimation of the pressure time product due to this mathematical approach. This is clearly shown in Fig. 9, where the error of PTP is much larger for the esophageal-derived muscle pressure. Similar to the esophageal-derived muscle pressure, the accuracy decreases with the increase of inspiratory pressure, with the decrease of muscle effort and with the increase of viscoelasticity. These effects are a result of the pressure drop over the viscoelastic element that is not taken into account. Again, it is challenging to relate these findings to the current literature since no true comparison is available. However, since PTP depends on the esophageal-derived muscle pressure, it is likely that the errors in the esophageal-derived muscle pressure also cause errors in PTP.

The error of the work of breathing is also mainly affected by viscoelasticity, and also decreases with increasing inspiratory effort. PEEP and inspiratory pressure have a smaller influence on WOB than on the esophageal-derived muscle pressure and PTP in the model as can be seen from Figure 6, Figure 7. It should be noted that while the high inspiratory pressure in Fig. 7 leads to a decrease in the error of the WOB, this might not always be the case. When the ‘Q=0’ line starts to lie beyond the chest wall pressure-volume line at very high inspiratory pressure (see Fig. 5), the calculation of WOB might not be valid anymore. Nevertheless, from Fig. 9, it can be seen that WOB has the lowest median error when compared to the other two esophageal pressure-based techniques. Comparing the found results to previous literature; in a clinical study [31], it was reported that the viscoelasticity is not taken into account by the Campbell diagram, confirming the results found in this study.

Finally, there are some practical considerations that have to be taken into account when employing estimation techniques relying on esophageal pressure. The estimates are continuous but require an obtrusive esophageal balloon catheter. The usage of such catheter requires highly trained staff, ventilators that are suited for an esophageal catheter, extra expenses for purchasing disposable catheters [47], and careful calibration in order to get an accurate estimate of the pleural pressure [48].

For the methods employing the occlusion, it was found that the error is independent of the viscoelasticity and patient effort strength since there is no flow and therefore no pressure drop over the viscoelastic element. The spread of the error and the outliers are much smaller in comparison with the other techniques.

The error observed during the occlusion does depend on the gas compressibility and the parasitic compliance of the tubing. The expansion of gas depends on FRC and FRC depends on PEEP. The error of the occlusion, therefore, does depend on the PEEP setting of the ventilator, which can be observed in Fig. 6. This finding implies that under the assumption that the respiratory mechanics of the patient do not change and while being in the same mode of ventilation, changes in the occlusion pressure are proportional to changes in the true muscle pressure, although absolute values are an underestimation depending on the expansion of gas. If the respiratory compliance and FRC, and parasitic compliance of the tubing are known, the occlusion pressure can be compensated using Equation (5) and the error will be very small. Equation (5) also explains the reason the error of the occlusion pressure is higher in Obese and ARDS archetype simulations; due to the smaller FRC and lower respiratory compliance, the effect of gas compressibility will be larger.

These findings in the model do correspond to the literature; it was already known that gas expansion has an influence on the occlusion pressure [49], [50], and it is even the main principle on which airway resistance and absolute lung volume determination are based [51], although it is not often reported.

The method based on the airway occlusion pressure seems a very promising and correct alternative since the accuracy of the whole breath occlusion is the highest among the evaluated techniques. The occlusion does not need obtrusive sensors, and does not depend on the viscoelasticity of the patient, but cannot be used to monitor continuously since the airflow temporarily needs to be halted. Nevertheless, it is unclear how pendelluft, autoPEEP and the changed muscle-force relations affect the accuracy of the occlusion-based techniques. AutoPEEP is also included in the model, but was not separately addressed. The influence of pendelluft and the changed muscle-force relations are hypotheses and are difficult to quantify by experiments.

During the measurement of $P_{0.1}$, the ratio in Equation (5), is also valid. However, the error in $P_{0.1}$ is larger as compared to the whole breath occlusion pressure, since $P_{0.1}$ is more sensitive to a phase shift between the pressure and flow signal [33]. This phase shift and autoPEEP also cause an underestimation since the airway pressure is delayed as compared to the muscle pressure. $P_{0.1}$ can still be useful for detecting changes in the respiratory effort; however, the value often does not reflect the true muscle effort at 100 ms. The findings on the magnitude of the error by $P_{0.1}$ and $\Delta P_{occ}$ agree with previous findings obtained in bench studies employing a lung simulator [52], [53] and on healthy subjects [54].

There are many more methods that rely on estimating the patient effort, resistance, and compliance of the respiratory system, or ventilator assistance based on the simplified equation of motion [55], [8], [9], which might all suffer from the same errors as presented here.

### 4.1 Limitations and future work

This study has a number of limitations:

1. So far, we only did an in silico comparison of the methods. The errors that we found are a result of our modeling choices, and should still be confirmed in clinical studies. Nevertheless, we expect that the order of magnitude of the errors is correct since the model is based on experimental data of the three patient groups, and all extra effects that are included in the model are reported in clinical measurements in the literature and will have an effect on the accuracy.
2. Even the non-linear one-lung model is a major simplification of the complex and heterogeneous lungs and airways, especially in patients with ARDS. Different from our model assumption, the pleural pressure is not homogeneous but depends on the location in the thorax. The influence of more complex, heterogeneous processes on the error of the inspiratory effort estimation could not be assessed.
3. It has previously been reported that pendelluft and the force-velocity relation of the muscle could have an impact on the accuracy of an occlusion [33]. Furthermore, concerns exist that a patient might modify a breath during a full breath occlusion [33]. Since limited data exist about these phenomena and they are difficult to quantify, we were not able to model the effect on the accuracy, and therefore did not take this into account.
4. The response of the patient to being ventilated is not included in the model. The change in the effort that is usually observed with increasing inspiratory pressure and PEEP was not modeled.
5. The model for the ventilator is overly simplistic. The focus of the ventilator model is on the breathing set tube impedance, which is sufficient to model the pressure at the airway opening, but might introduce differences. In a mechanical ventilator, there is usually a control algorithm to regulate the flow and pressure, which has an influence on the waveform. This also depends on the brand of ventilator that is used and is therefore difficult to incorporate.
6. No measurement noise and cardiac oscillations were added to the pleural pressure and airway pressure. Usually, these will distort the pressures and may increase the calculated error even further. However, the amount will depend on the patient, measurement setup, the amount of mucus in the breathing set, and will likely affect the estimation techniques in a similar way. Therefore, these signals are not included in the simulations.

The model used in this paper is too difficult to use in the clinic for the estimation of the inspiratory effort since the model contains too many parameters and suffers from identifiability issues when only the pressure, flow, and tidal volume are recorded. In future work, our idea would be to develop a simplified version of the model with fewer parameters that can be determined in the clinic, while still retaining the most important features neglected by the linear model.

## 5 Conclusion

In this paper, we evaluated the accuracy of different techniques for the estimation of muscle effort during mechanical ventilation, using a previously validated simulation dataset as ground truth. During the simulations, all pressure-based methods underestimate the true muscle effort in the presence of viscoelasticity and gas compression. This is especially important for ARDS and obese patients since the viscoelasticity and effect of gas compressibility are higher. The error still needs to be evaluated and validated in clinical studies. For the techniques based on the esophageal pressure, the viscoelasticity is the main reason for the error in the model, and the exact error depends on the ventilator settings, inspiratory effort strength, and respiratory mechanics. For the occlusion-based methods, the error mostly depends on the gas compressibility and is independent of the patient's effort strength. The findings in the study provide important clinical insights into lung- and diaphragm-protective mechanical ventilation.

## Funding statement

This research did not receive any specific grant from funding agencies in the public, commercial, or not-for-profit sectors.

## CRediT authorship contribution statement

Anouk van Diepen; Pierre Woerlee: Conceived and designed the experiments; Performed the experiments; Analyzed and interpreted the data; Contributed reagents, materials, analysis tools or data; Wrote the paper.

Tom Bakkes; Ashley De Bie; Simona Turco: Conceived and designed the experiments; Analyzed and interpreted the data; Wrote the paper.

Arthur Bouwman: Conceived and designed the experiments.

Massimo Mischi: Conceived and designed the experiments; Wrote the paper.

## Declaration of Competing Interest

The authors declare no competing interests.

## Data Availability

Data will be made available on request.

## Appendix A Supplementary material

MMC Used model parameters and detailed RMSPE information. MMC
