# Supplementary material for: Evaluation of the accuracy of established patient inspiratory effort estimation methods during mechanical support ventilation
Source: Heliyon. 2023 Feb 10;9(2):e13610. doi: 10.1016/j.heliyon.2023.e13610 (PMC9958297; doi:10.1016/j.heliyon.2023.e13610)
Supplement: MMC — Used model parameters and detailed RMSPE information. [file mmc1.pdf]

Supplementary material

December 13, 2022

1 Parameters for the model of the respiratory system

Table 1: Parameters used for the different patient archetypes patient 1

	Healthy	Obese	ARDS
RV (L)	1.24	0.25	0.25
TLC (L)	5.19	4.2	4.2
Acw (cmH_2O)	1.4	4.0	1.4
Bcw (cmH_2O)	-3.5	-3.5	-3.5
Al (cmH_2O)	9.98	4.0	2.85
Bl (cmH_2O)	0.1	0.1	0.10
Dl	17	11.2	10.0
As ($cmH_2O/(L.s)$)	2.2	3.00	4.0
Bs ($cmH_2O/(L.s)$)	0.50	0.50	3.2
Ks	-10.9	-5.00	-5.0
Vstar (L)	5.3	4.4	4.31
Vcmax (L)	0.1	0.07	0.055
Ac (cmH_2O)	0.341	0.2	0.16
Bc (cmH_2O)	9.692	10	10
Dc	0.411	0.411	0.411
Kc	0.21	0.40	0.4
Au ($cmH_2O/(L.s)$)	0.34	2.0	0.34
Ku ($cmH_2O/(L^2.s^2)$)	0.46	8.0	0.46
Rve ($(cmH_2O.s)/L$)	1.0	18.0	3.0
Cve (L/cmH_20)	0.5	0.2	0.2

Table 2: Parameters used for the different patient archetypes patient 2

	Healthy	Obese	ARDS
RV (L)	2.04	0.3	0.41
TLC (L)	8.27	6.10	6.6
Acw (cmH_2O)	-3.1	4.2	-3.1
Bcw (cmH_2O)	-3.5	-4.5	-3.5
Al (cmH_2O)	8.27	5.90	4.5
Bl (cmH_2O)	0.172	0.12	0.04
Dl	6.9	10	10
As ($cmH_2O/(L.s)$)	2.8000	3.8182	5.0909
Bs ($cmH_2O/(L.s)$)	0.0200	0.0200	0.1280
Ks	-8.9900	-4.1239	-4.1239
Vstar (L)	10.3000	8.5509	8.8619
Vcmax (L)	0.1250	0.0875	0.0688
Ac (cmH_2O)	0.1884	0.1105	0.0884
Bc (cmH_2O)	12.7200	13.1242	13.1242
Dc	0.411	0.411	0.411
Kc	0.4900	0.9333	1.8667
Au ($cmH_2O/(L.s)$)	0.3100	1.8235	0.3100
Ku ($cmH_2O/(L^2.s^2)$)	0.4000	6.9565	0.4000
Rve ($(cmH_2O.s)/L$)	1	18	3
Cve (L/cmH_20)	0.5	0.2	0.15

Table 3: Parameters used for the different patient archetypes patient 3

	Healthy	Obese	ARDS
RV (L)	1.61	0.32	2.522
TLC (L)	5.4	5.04	5.04
Acw (cmH_2O)	-0.5	3.811	-0.5
Bcw (cmH_2O)	-3.5	-3.5	-3.5
Al (cmH_2O)	8	5	3.48
Bl (cmH_2O)	0.0756	0.09	0.06
Dl	17	14	15.3
As (cmH_2O/(L.s))	2.4700	3.3682	4.4909
Bs (cmH_2O/(L.s))	0.0200	0.0200	0.1280
Ks	-6.5000	-2.9817	-2.9817
Vstar (L)	8.4100	6.9819	6.839
Vcmax (L)	0.1647	0.1153	0.0906
Ac (cmH_2O)	0.1331	0.0781	0.0625
Bc (cmH_2O)	15.7500	16.2505	16.2505
Dc	0.411	0.41	0.411
Kc	0.3200	0.6095	1.5238
Au (cmH_2O/(L.s))	0.3100	1.8235	0.3100
Ku ($cmH_2O/(L^2.s^2)$)	0.3200	5.5652	0.4174
Rve ((cmH_2O.s)/L)	1	18	3
Cve (L/cmH_20)	0.5	0.2	0.15

Table 4: Parameters used for the different patient archetypes patient 4

	Healthy	Obese	ARDS
RV (L)	1.91	0.38	0.38
TLC (L)	7.2	5.7	5.7
Acw (cmH_2O)	0.7	3.811	0.7
Bcw (cmH_2O)	-3.5	-3.5	-3.5
Al (cmH_2O)	9.88	5.5	3.95
Bl (cmH_2O)	0.1	0.07	0.05
Dl	15	15	12
As (cmH_2O/(L.s))	5.4700	7.4591	7.4591
Bs (cmH_2O/(L.s))	0.0200	0.0200	0.1280
Ks	-5.1300	-2.3532	-2.3532
Vstar (L)	7.3700	6.1185	5.9933
Vcmax (L)	0.1000	0.0700	0.0550
Ac (cmH_2O)	0.0970	0.0569	0.0455
Bc (cmH_2O)	18.8500	19.4490	7.7796
Dc	0.411	0.411	0.411
Kc	0.2400	0.4571	0.2286
Au (cmH_2O/(L.s))	0.3100	1.8235	0.3100
Ku ($cmH_2O/(L^2.s^2)$)	0.2000	3.4783	0.2000
Rve ((cmH_2O.s)/L)	1	18	3
Cve (L/cmH_20)	0.5	0.2	0.15

2 Parameters for the ventilator

The equations for the Rohrer's resitances in the ventilator model (Fig. 1) are given by the equations:

$$R_{et} = A_t + K_t \dot{V}_{et} \quad (1)$$

$$R_{t,insp} = A_{ti} + K_{ti} \dot{V}_{ti} \quad (2)$$

$$R_{t,exp} = A_{te} + K_{te} \dot{V}_{te} \quad (3)$$

$$(4)$$

The parameters used during the simulations are given in Table 5.

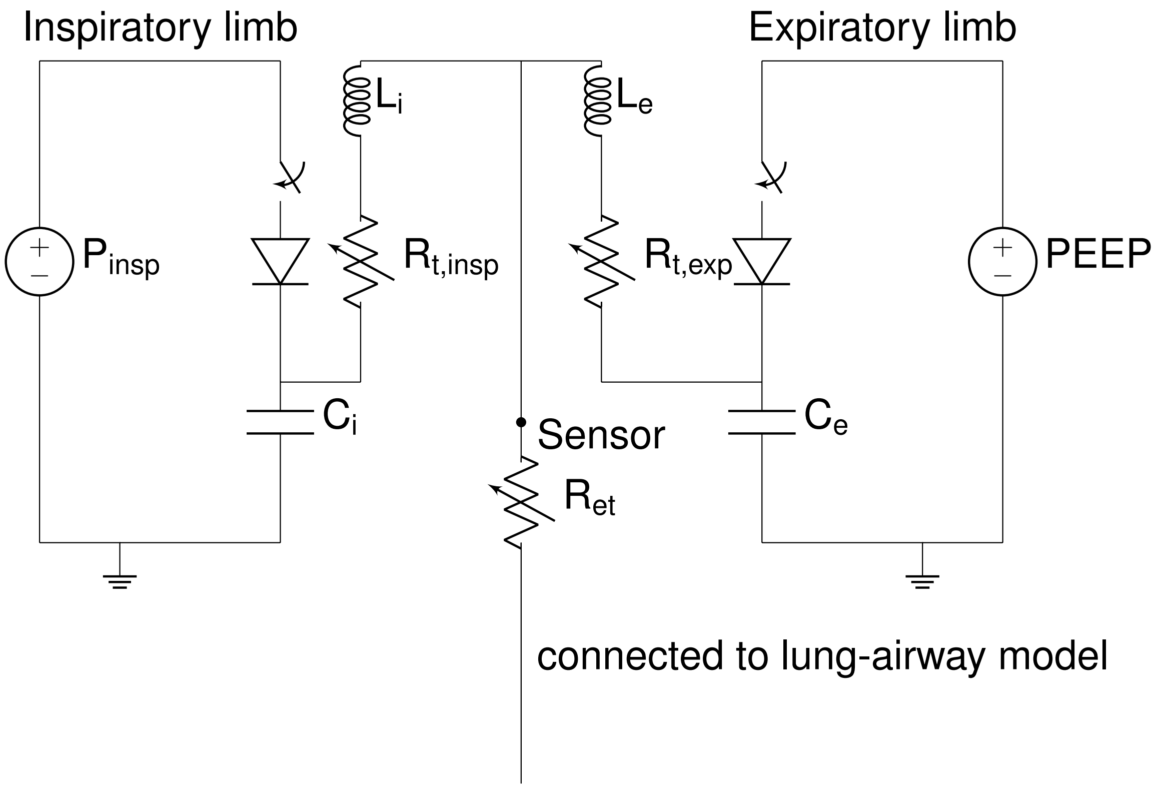

Figure 1: Equivalent circuit of the ventilator model.

Table 5: Parameters used for the ventilator

Parameter	Value
Ati ($cmH_2O/(L.s)$)	0.5
Kti ($cmH_2O/(L^2.s^2)$)	2.3
Ate ($cmH_2O/(L.s)$)	0.5
Kte($cmH_2O/(L^2.s^2)$)	2.3
Li ($cmH_2O.s^2/L$)	0.08
Le ($cmH_2O.s^2/L$)	0.08
At ($cmH_2O/(L.s)$)	1
Kt ($cmH_2O/(L^2.s^2)$)	2
Ci ($(cmH_2O.s)/L$)	0.0015
Ce ($(cmH_2O.s)/L$)	0.0015

3 Figures esophageal-derived muscle pressure

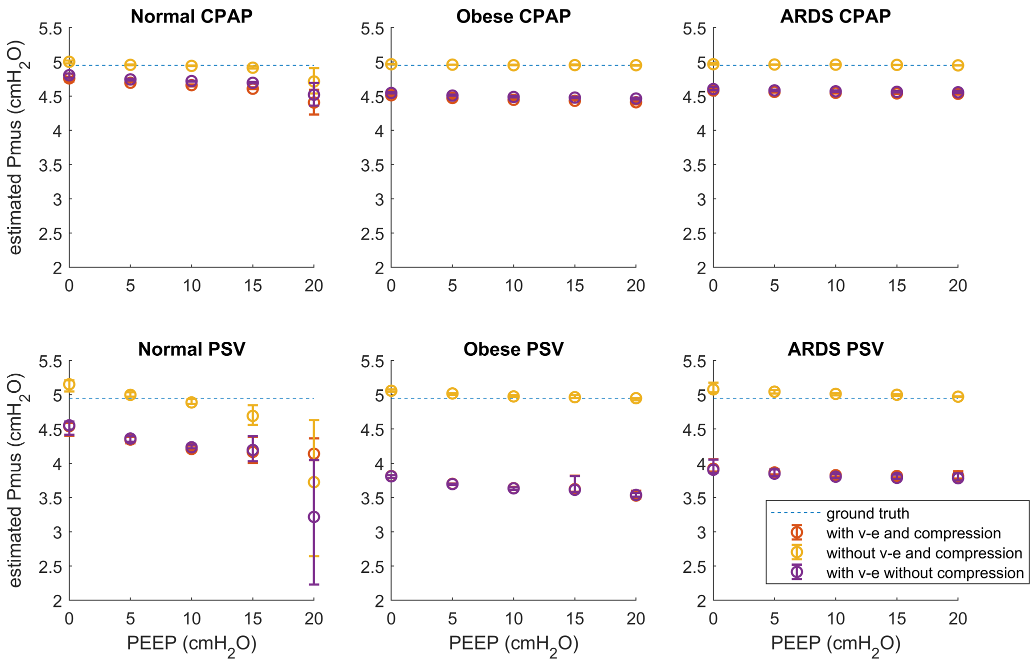

Figure 2: The swing in the esophageal-derived muscle pressure calculated for CPAP and PSV breaths including and excluding the viscoelasticity and compression. The experiment employed parameters corresponding to four healthy persons, four obese, four ARDS. The ground truth swing muscle pressure was 4.95 cmH_2O. PS = PEEP+10 cmH_2O for PSV.

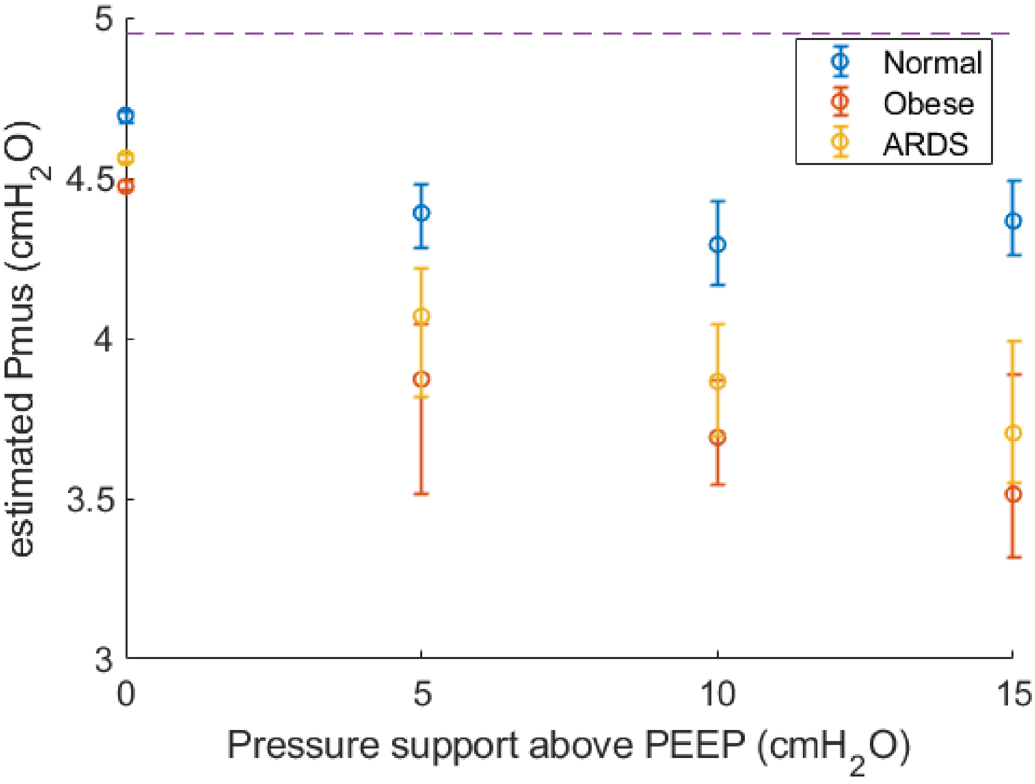

Figure 3: The evolution of the esophageal-derived muscle swing as function of the pressure support level with PEEP=5 cmH_2O. The ground truth swing muscle pressure was 4.95 cmH_2O.

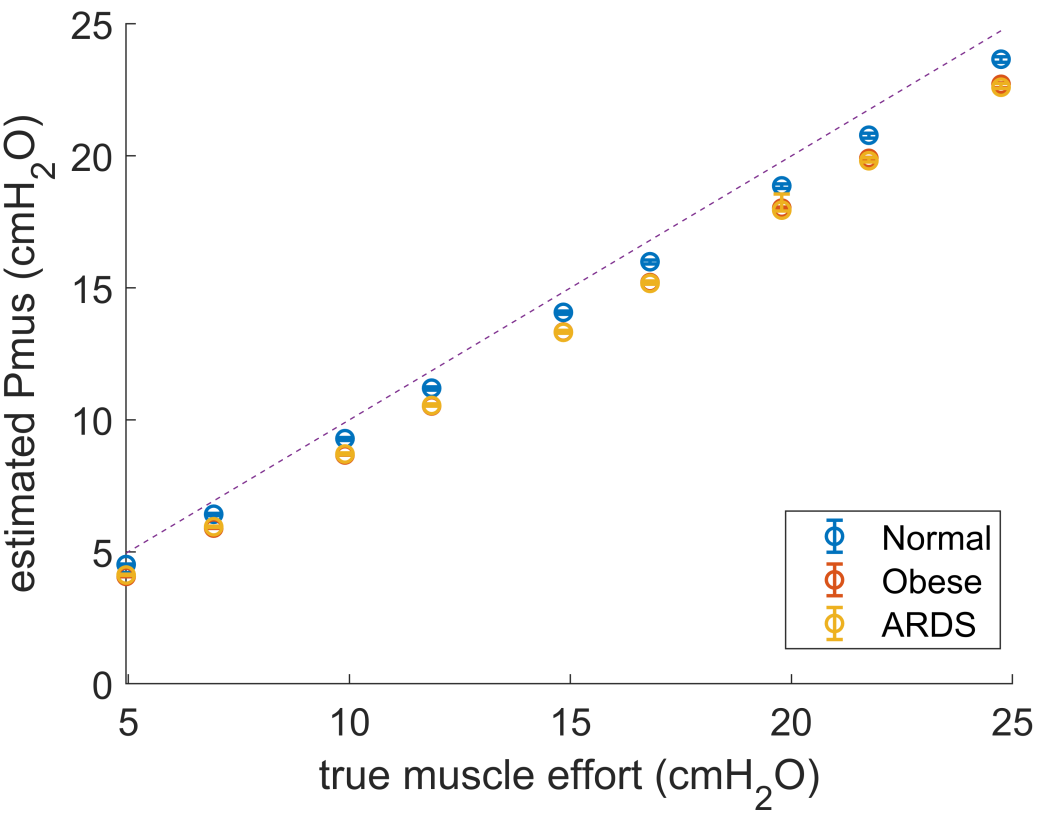

Figure 4: The evolution of the esophageal-derived muscle swing as function of the patient effort with PEEP=5 cmH_2O PSL = PEEP+5 cmh2O.

4 Figures pressure time product

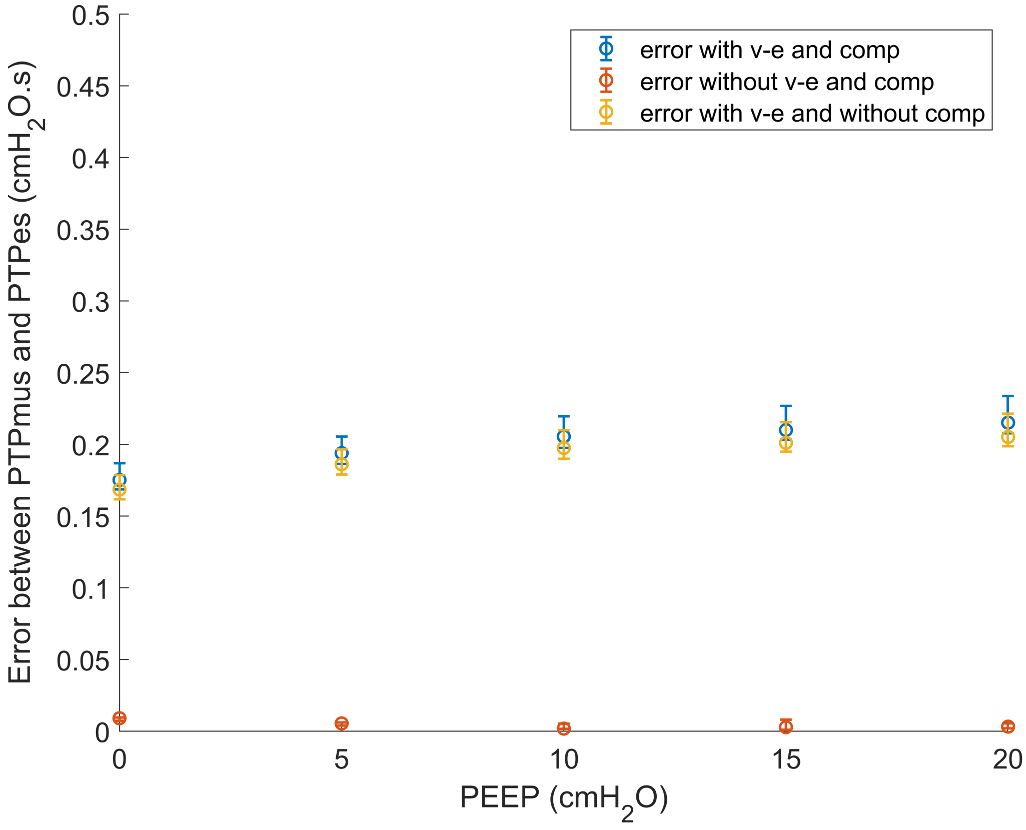

Figure 5: The error between PTPmus and PTPes when visco-elasticity and compression is included, when they are both excluded, and when visco-elasticity is included but compression is excluded for CPAP breaths for an 'obese' archetype with Pmus = 4.95 cmH_2O.

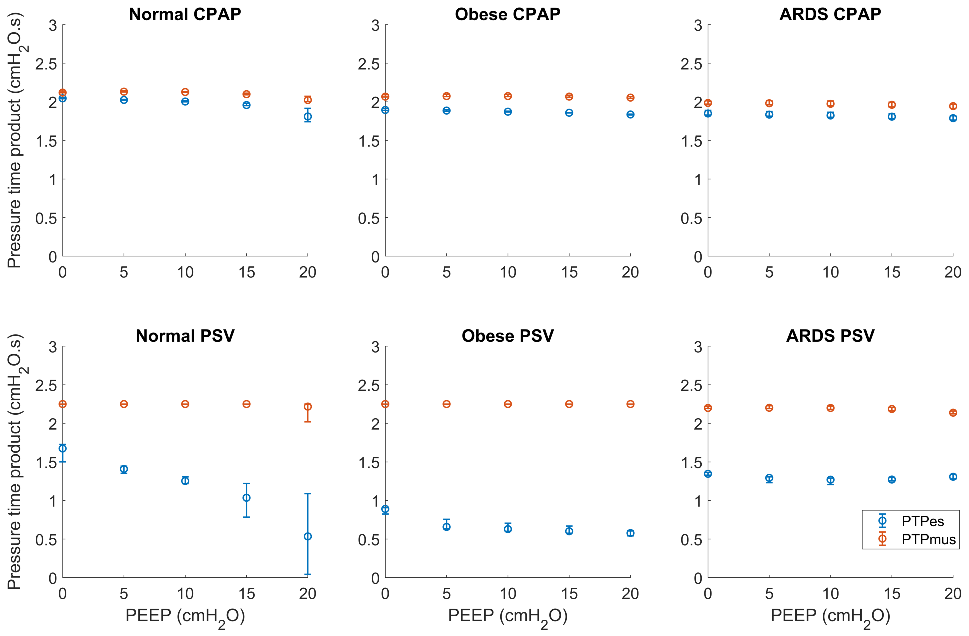

Figure 6: The esophageal-derived PTP for CPAP and PSV for one breath, and the true muscle-derived PTP for CPAP and PSV (the ground truth), both including visco-elasticity and compression. During pressure support PSL=PEEP+15 cmH_2O.

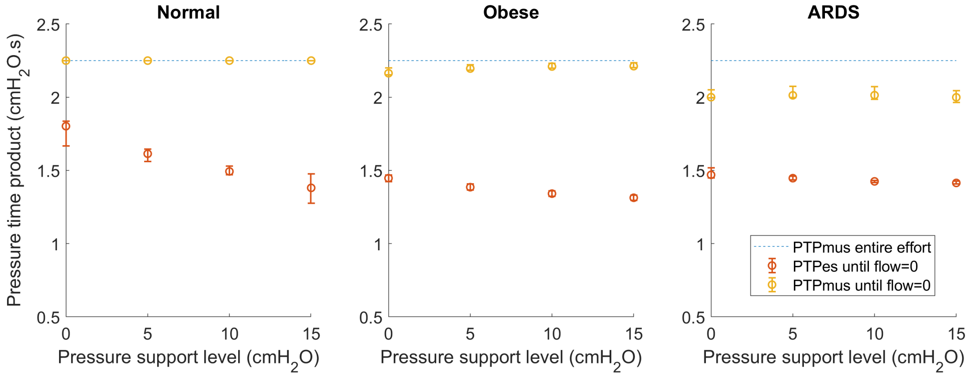

Figure 7: PTP for PEEP = 5 cmH_2O, P_{mus}= 4.95 cmH_2O, and increasing levels of pressure support for different patient archetypes. Note that the patient effort may continue after the flow becomes zero. For this reason, the exact value of PTP_{mus} over the entire effort is higher than PTP_{mus} over the inspiratory time only.

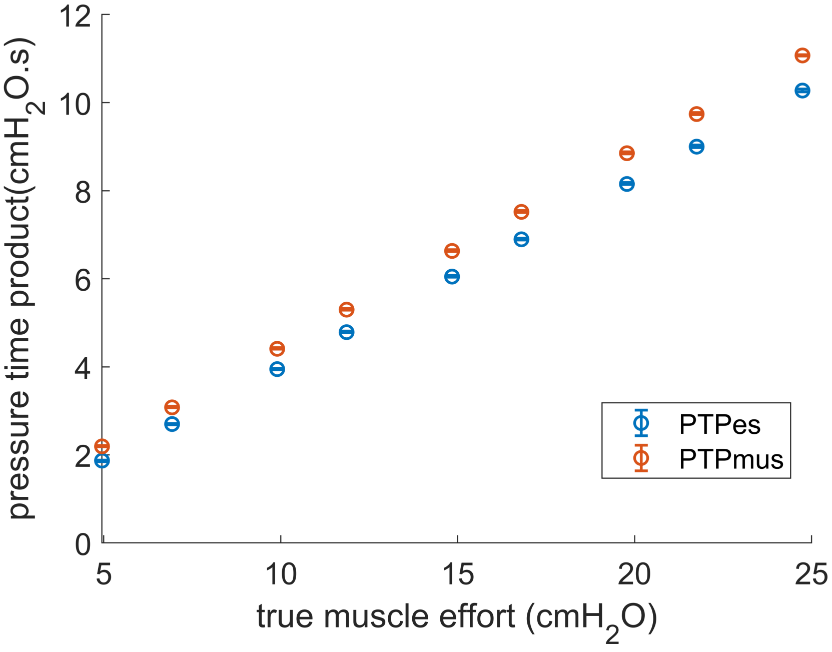

Figure 8: PTP for PSV with PEEP = 5 cmH_2O and PSL = PEEP+5 cmH_2O with increasing effort for four normal archetype patients.

5 Work of breathing

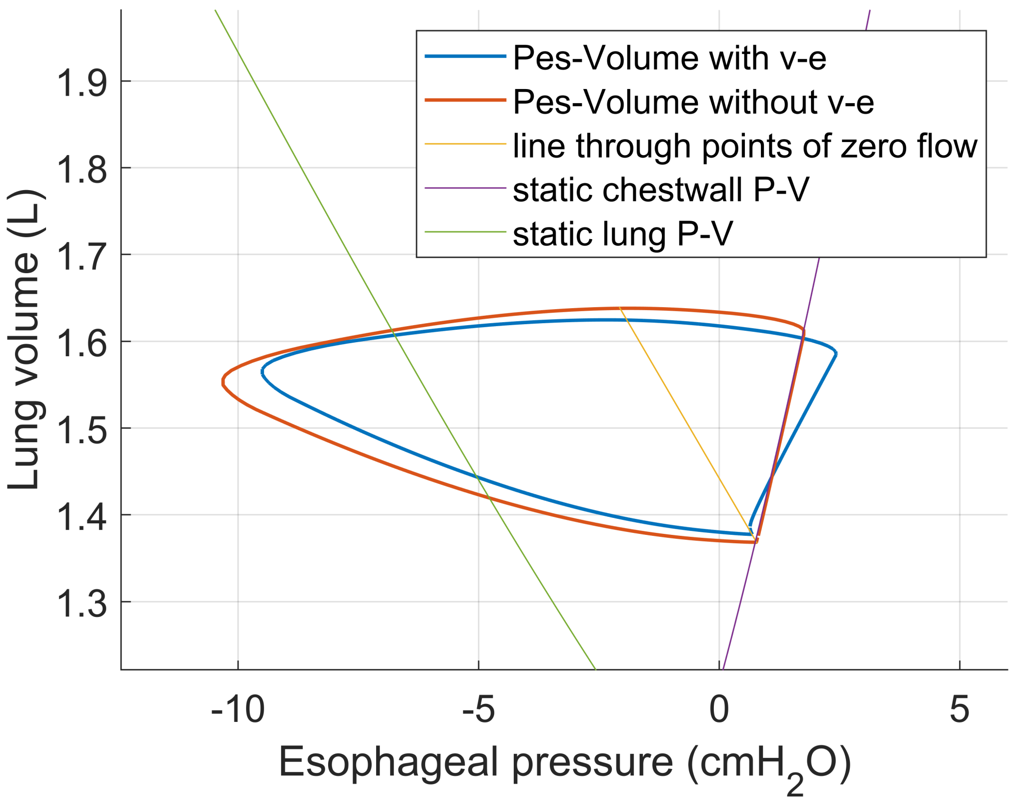

Figure 9: The simulated Campbell diagram during CPAP with PEEP= 5 cmH_2O and the maximum patient effort is -11.85 cmH_2O for an 'obesity' archetype simulation. The blue curve is a simulation that includes viscoelasticity, while the orange curve is a simulation in which viscoelasticity is omitted.

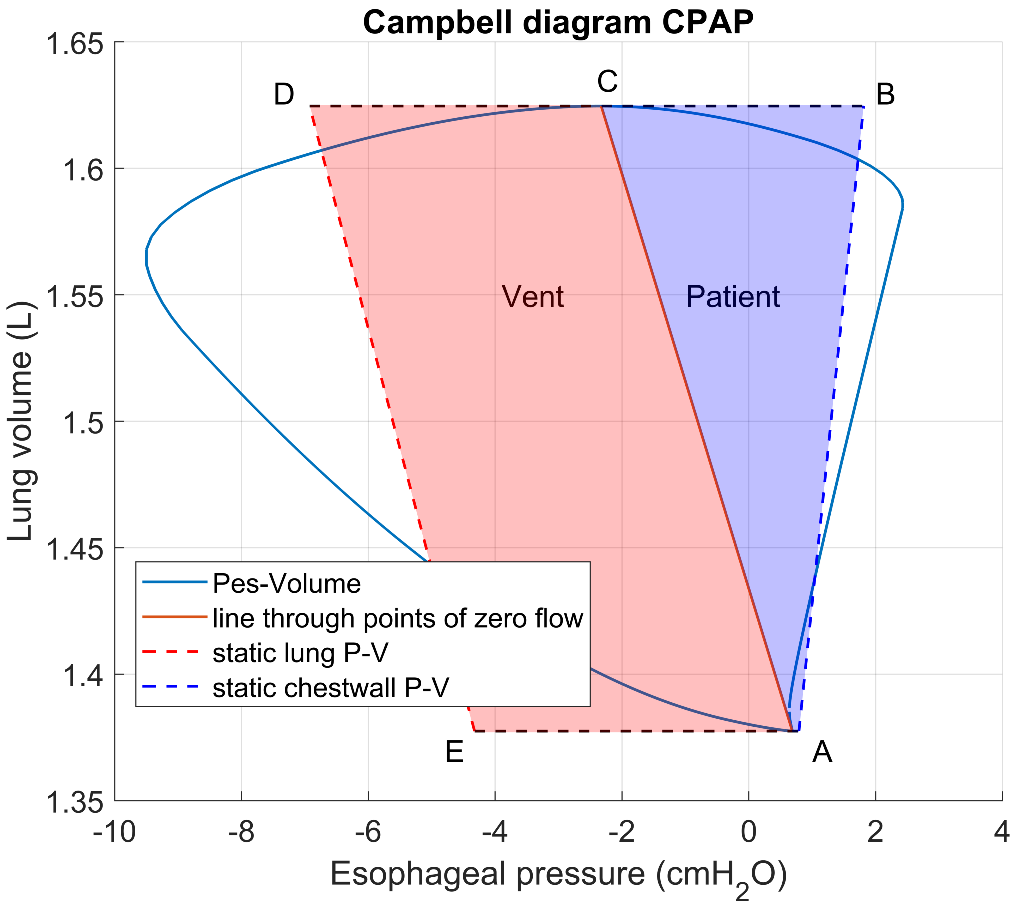

Figure 10: The simulated Campbell diagram during CPAP with PEEP= 5 cmH_2O and the maximum patient effort is -11.85 cmH_2O for an ‘obesity’ archetype simulation. The red area shows the elastic work done by the ventilator, while the blue area shows the elastic work done by the patient. Often only the elastic work done by the patient is considered.

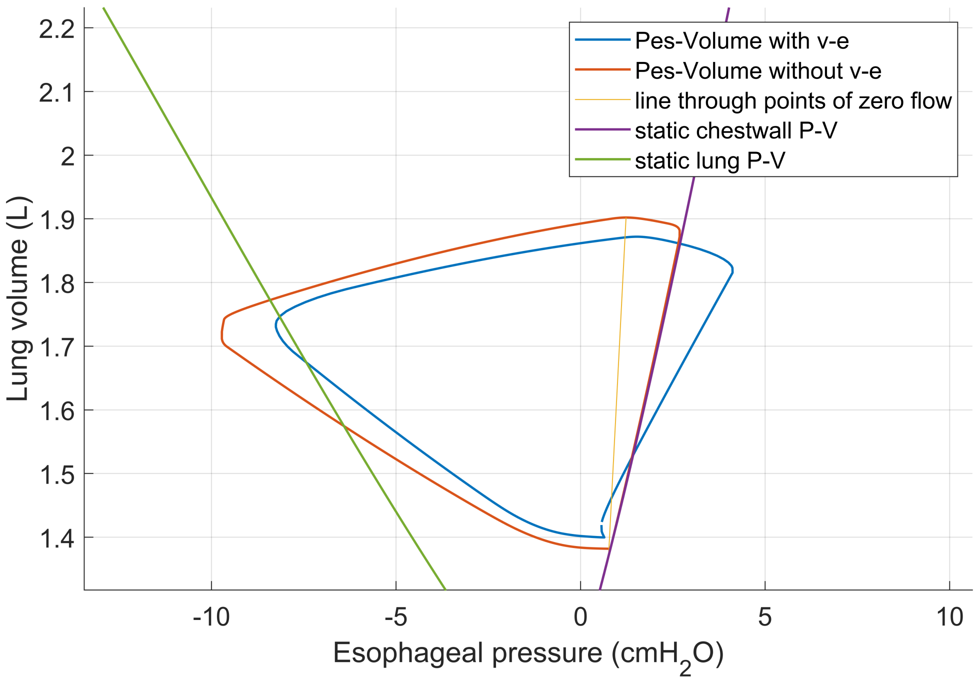

Figure 11: The simulated Campbell diagram during PSV with PEEP= 5 cmH_2O, PS= PEEP+10 cmH_2O and the maximum patient effort is -11.85 cmH_2O for an ‘obesity’ archetype simulation. The blue curve is a simulation that includes viscoelasticity, while the orange curve is a simulation in which viscoelasticity is omitted.

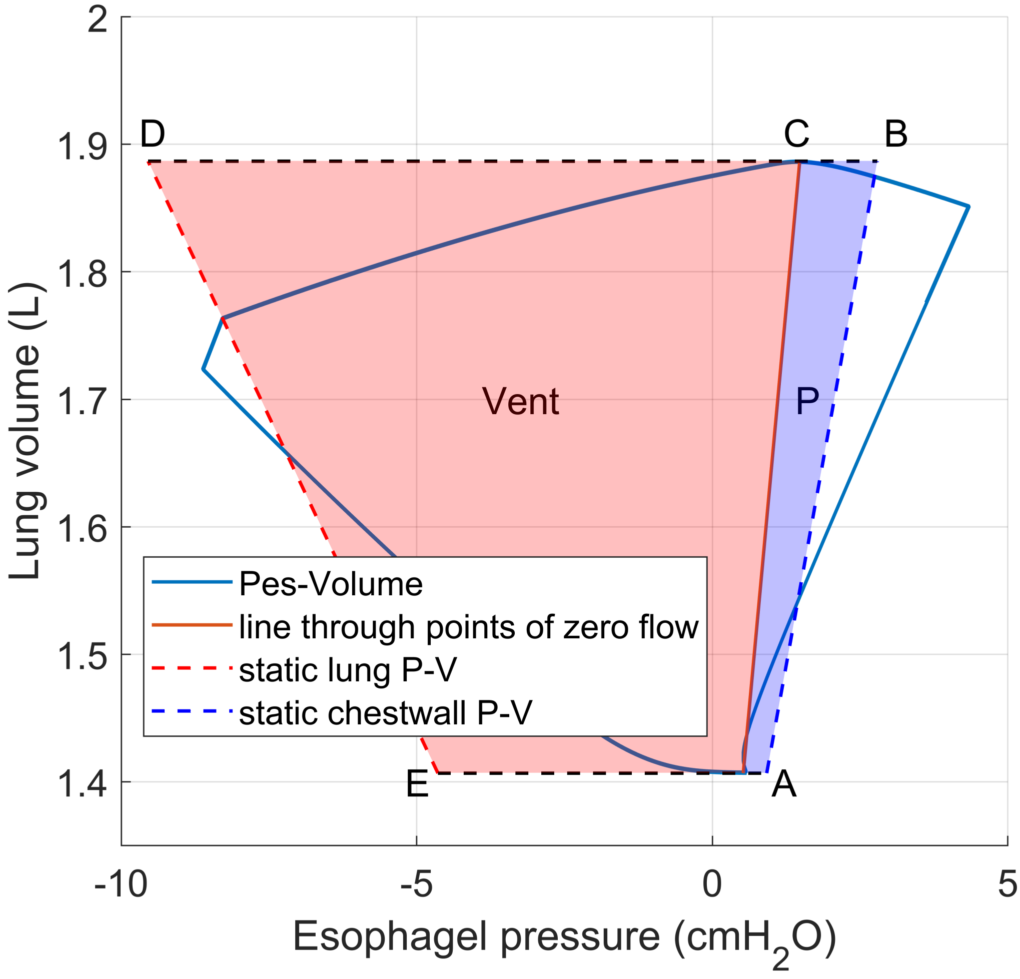

Figure 12: The simulated Campbell diagram during PSV with PEEP= 10 cmH_2O, PS= PEEP+10 cmH_2O and the maximum patient effort is -11.85 cmH_2O for an 'obesity' archetype simulation.

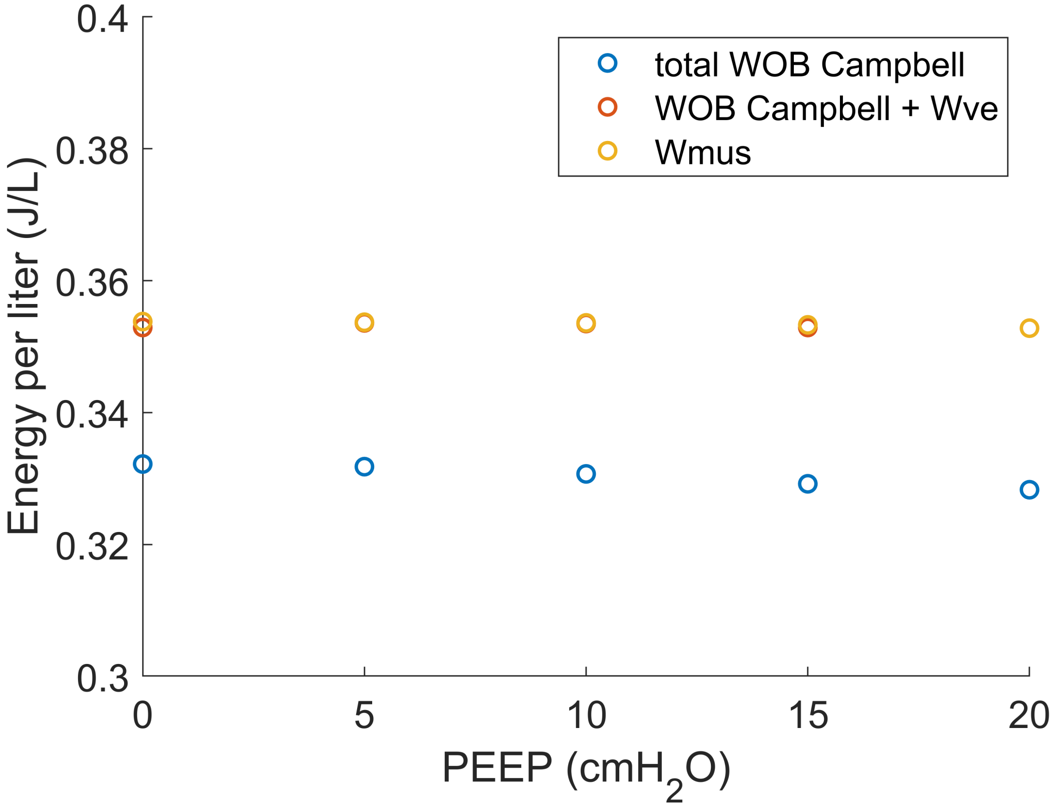

Figure 13: Comparison of the total WOB calculated by the Campbell diagram (elastic WOB derived by the Campbell diagram + resistive WOB derived by the Campbell diagram), the total WOB derived by the Campbell diagram plus the energy dissipated in the viscoelastic element (W_{ve}), and the total patient work derived by the integral of P_{mus} (W_{mus}) for an obese-archetype parameter set during CPAP with P_{mus} = -4.95 cmH_2O.

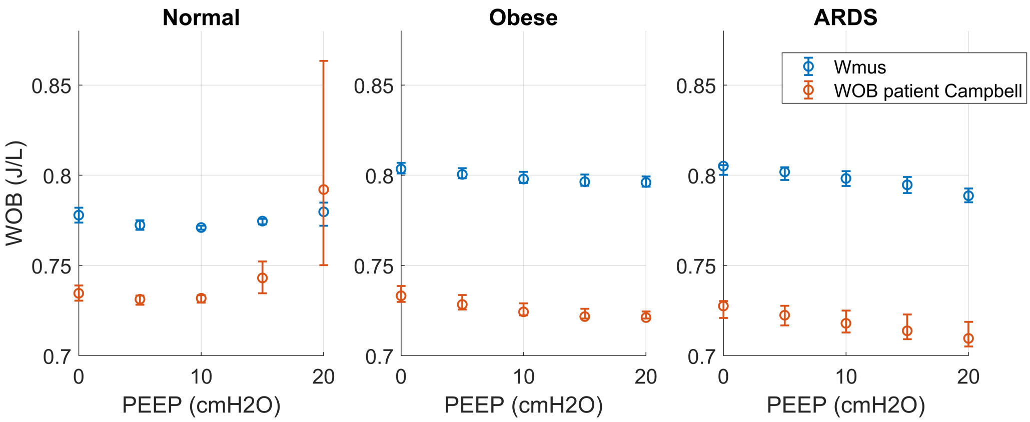

Figure 14: WOB as calculated by the Campbell diagram and Wmus during PSV with PS = PEEP+15 cmH_2O and Pmus = 4.95 cmH_2O.

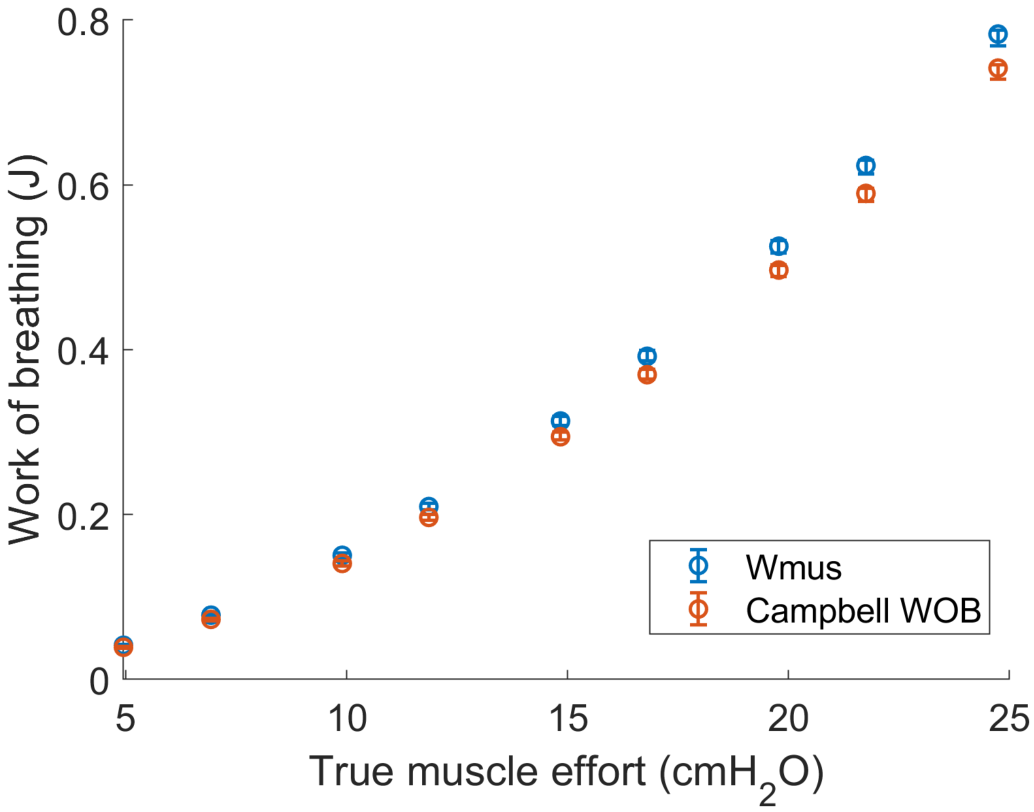

Figure 15: WOB as calculated by the Campbell diagram and Wmus for obese patient archetypes with increasing inspiratory effort during CPAP. PEEP is fixed to 5 cmH_2O.

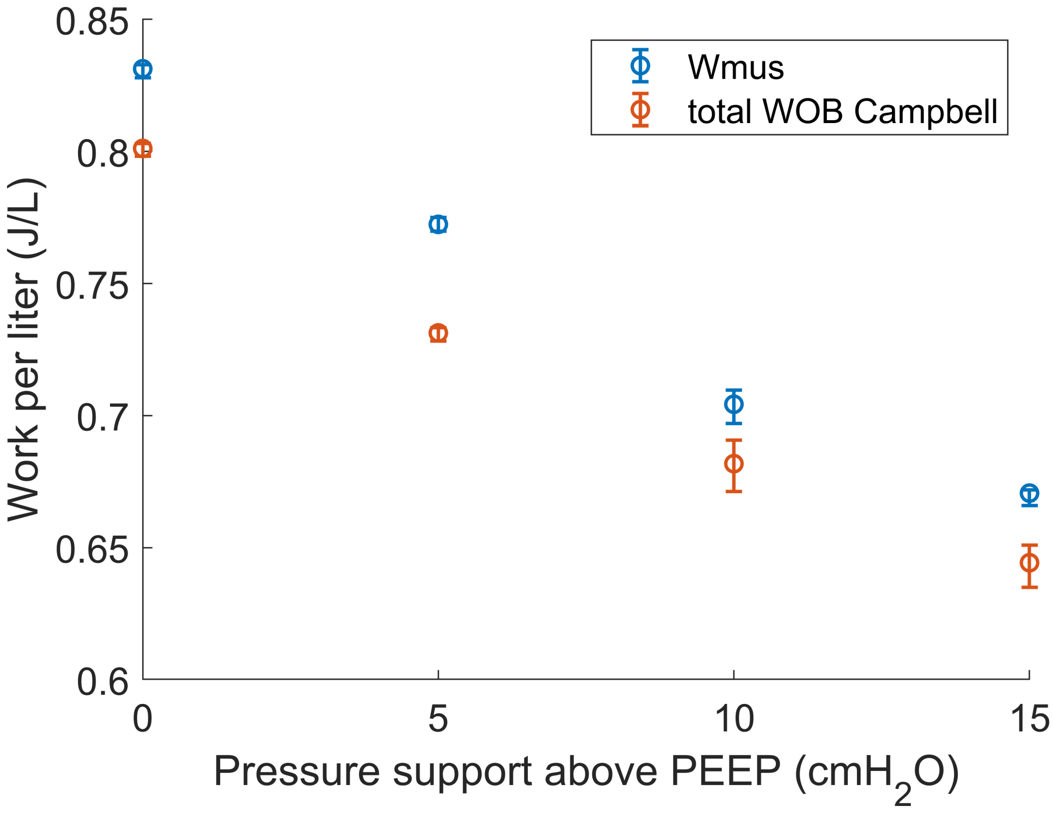

Figure 16: Total WOB and Wmus for normal archetype simulations when PSL is increased. PEEP is fixed to 5 cmH_2O.

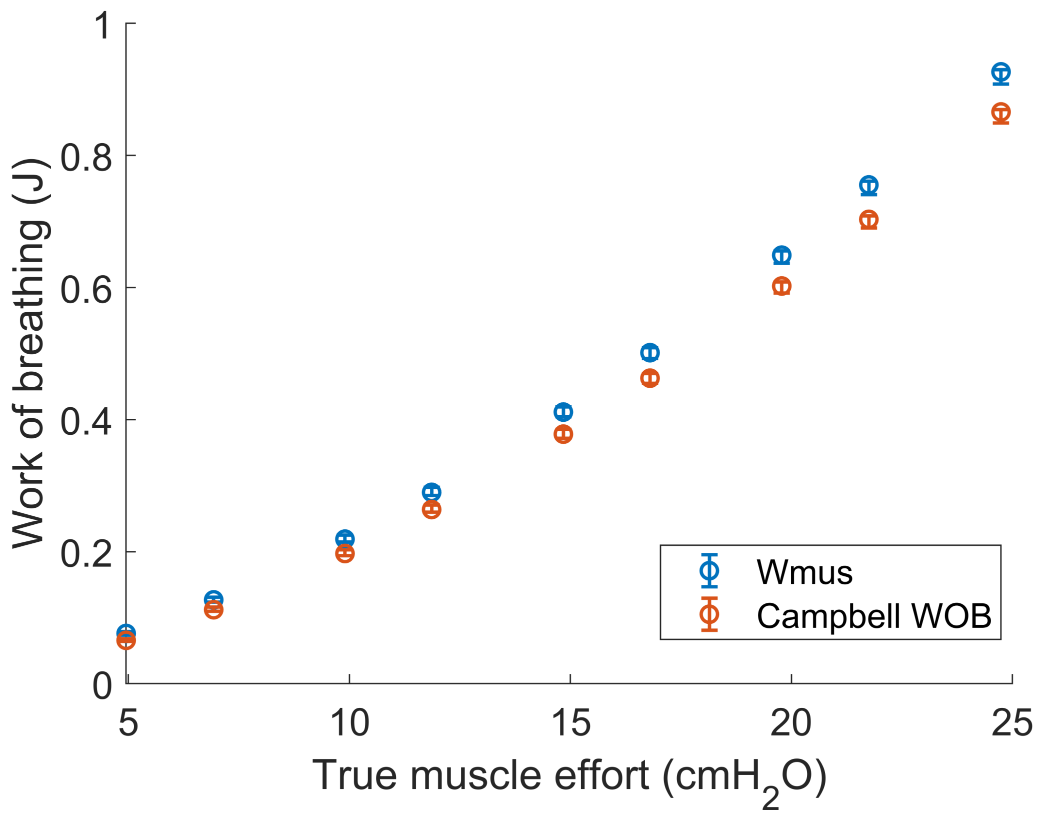

Figure 17: Total WOB and Wmus for obese archetype simulations when patient effort is increased during PSV. PEEP is fixed to 5 cmH_2O and PSL = PEEP + 5 cmH2O.

6 Occlusion pressure

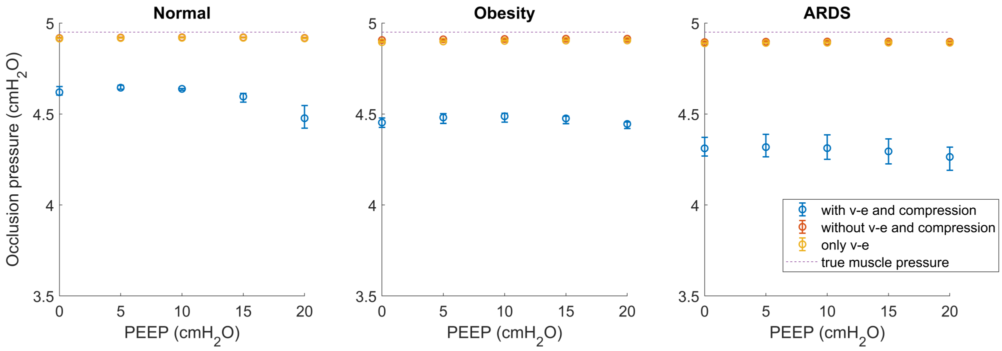

Figure 18: The swing in the airway opening pressure during an occlusion (ΔP_{occ}) with viscoelasticity and gas compression (blue), without viscoelasticity and gas compression (orange), and with viscoelasticity but without gas decompression (yellow) for different patient archetypes. $P_{mus} = 4.95$ cmH_2O, while PEEP is varied and pressure support is not present.

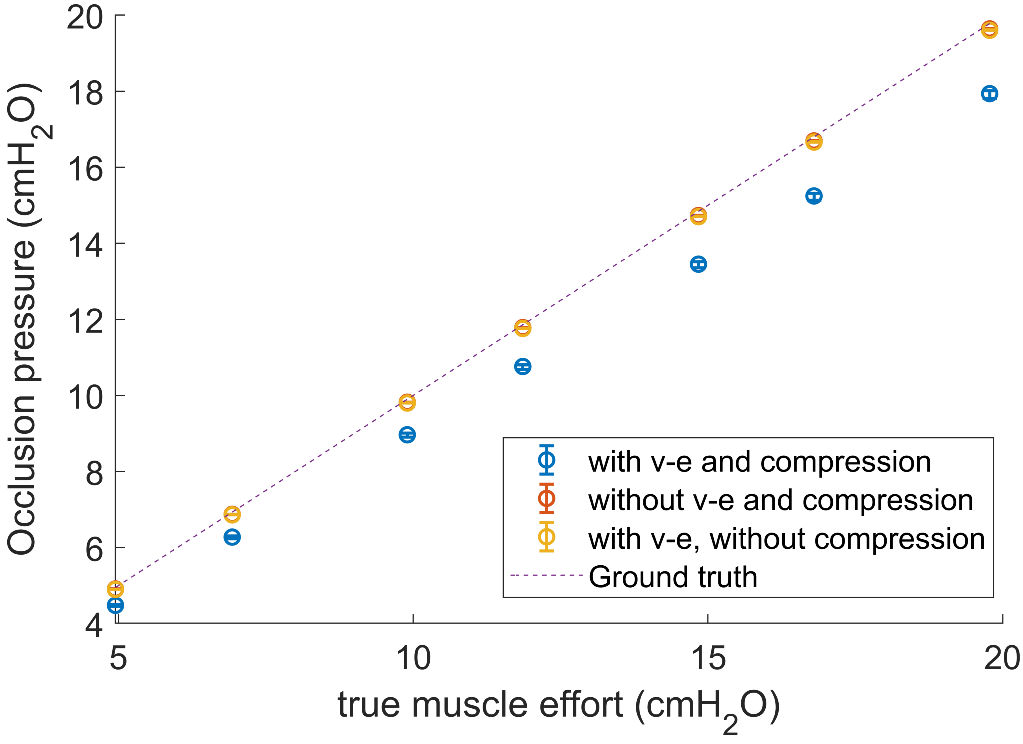

Figure 19: Occlusion pressure for an obese-archetype with PEEP=5 cmH_2O and increasing inspiratory muscle effort.

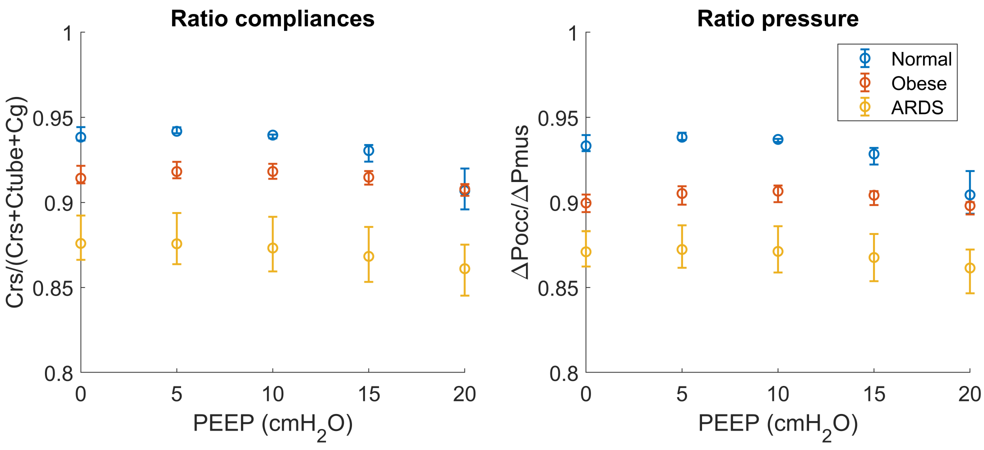

Figure 20: The ratio of the compliances ($C_{rs}/(C_{rs}+C_{tube}+C_g)$) for different patient archetypes, compared to the pressure ratio ($\Delta P_{occ}/\Delta P_{mus}$) during an occlusion.

7 P0.1

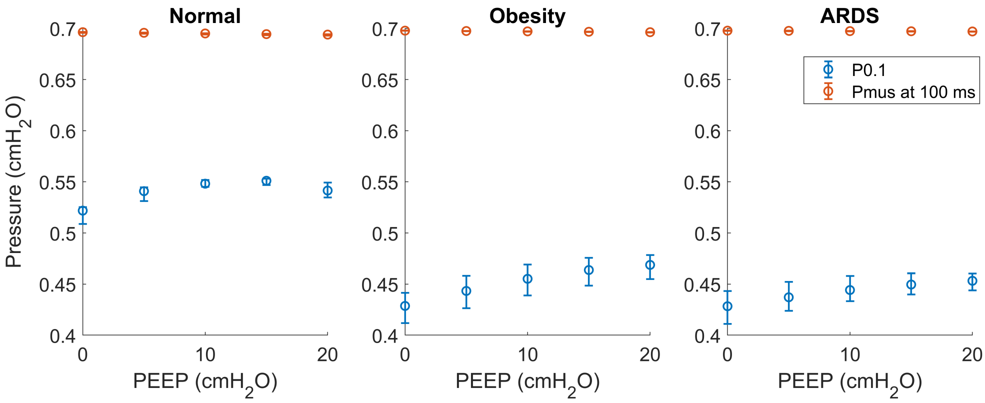

Figure 21: P0.1 as measured at the airway opening during an occlusion compared to the true pressure drop in muscle pressure after 100 ms including visco-elasticty and compression and excluding visco-elasticity and compression.

8 Overall results

Table 6: RMSPE, minPE, and maxPE for the different techniques including gas compression and viscoelasticity for P_{mus}= 4.95, 9.9, 14.85, 19.78 cmH_2O, PEEP = 0, 5, 10, 15, 20 cmH_2O, and PS = 0, 5, 1 0, 15 cmH_2O above PEEP.

	Normal RMSPE (minPE-maxPE)	Obese RMSPE (minPE-maxPE)	ARDS RMSPE (minPE-maxPE)
Esophageal derived P_{mus}	12.1% (0%-49%)	16.4% (6.6%-36.0%)	13.7% (4.9%-30.3%)
PTP_{es}	20.7% (0%-100.8%)	27% (6.7%-76.7%)	18.5% (4.6%-48.8%)
WOB	12.4% (0.02%-87.5%)	9.3% (0.9%-23.8%)	14.4% (5.3%-34%)
ΔP_{occ}	7.1% (5.3%-11.7%)	9.9% (8.6%-11.1%)	13.2% (10.1%-16.5%)
$P_{0.1}$	22% (2.7%-30.2%)	36% (30.2%-42.2%)	36.6% (33.3%-41.7%)

Table 7: RMSPE, minPE, and maxPE for the different techniques including viscoelasticity, but excluding gas compression for P_{mus}= 4.95, 9.9, 14.85, 19.78 cmH_2O, PEEP = 0, 5, 10, 15, 20 cmH_2O, and PS = 0, 5, 1 0, 15 cmH_2O above PEEP.

	Normal RMSPE (minPE-maxPE)	Obese RMSPE (minPE-maxPE)	ARDS RMSPE (minPE-maxPE)
Esophageal derived P_{mus}	12.3% (0%-93%)	16.1% (4.9%-37%)	13.8% (4.5%-32%)
PTP_{es}	20.5% (0%-107%)	27% (6.2%-80.3%)	18.7% (4.2%-49.8%)
WOB	12.4% (0.02%-87.5%)	9.3% (0.9%-23.8%)	14.4% (5.3%-34%)
ΔP_{occ}	2.9% (0.6%-4.2%)	5.3% (0.8%-7.4%)	7.2% (1.0%-9.0%)
$P_{0.1}$	12.3% (1.2%-19.6%)	22% (2.4%-31.3%)	22.6% (2.7%-30%)

Table 8: RMSPE, minPE, and maxPE for the different techniques excluding gas compression and viscoelasticity for P_{mus}= 4.95, 9.9, 14.85, 19.78 cmH_2O, PEEP = 0, 5, 10, 15, 20 cmH_2O, and PS = 0, 5, 1 0, 15 cmH_2O above PEEP.

	Normal RMSPE (minPE-maxPE)	Obese RMSPE (minPE-maxPE)	ARDS RMSPE (minPE-maxPE)
Esophageal derived P_{mus}	6.8% (0%-40%)	0.8% (0%-4.5%)	1.3% (0%-9.2%)
PTP_{es}	9.6% (0%-66%)	1.5% (0%-10%)	1.7% (0%-15.7%)
WOB	12.84% (0%-90.84%)	0.5% (0%-2.6%)	0.4% (0%-2.8%)
ΔP_{occ}	2.6% (0.5%-3.9%)	4.1% (0.6%-6.1%)	6.3% (0.9%-8.1%)
$P_{0.1}$	11.9% (1.1%-19.11%)	20.9% (1.9%-30%)	21.6% (2.5%-29%)

9 Effect of viscoelasticity

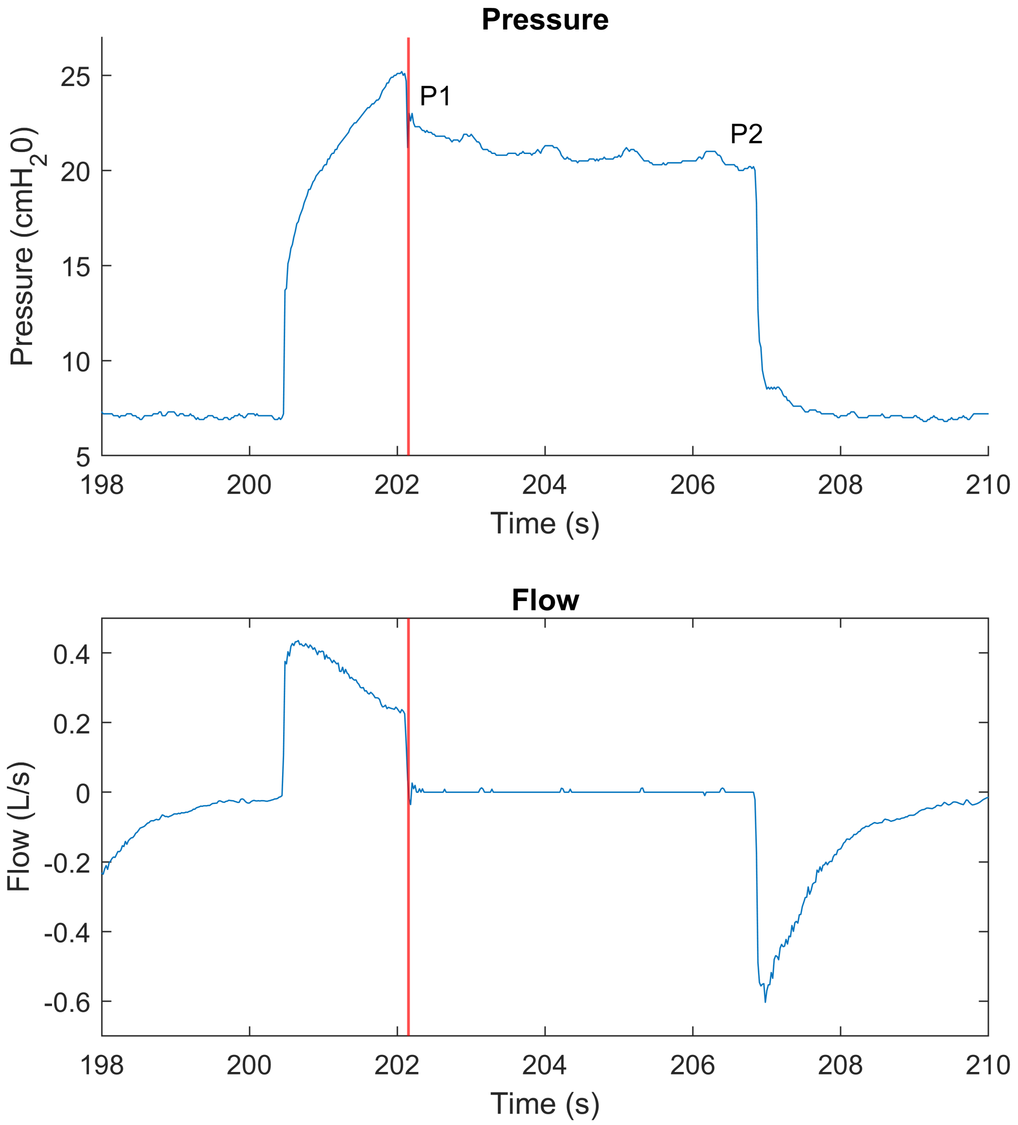

Figure 22: The difference in pressure between P1 and P2 can be explained as pendelluft or viscoelasticity.
